# Task completion without commitment

**DOI:** 10.1007/s10683-024-09824-2

**Published:** 2024-05-14

**Authors:** David J. Freeman, Kevin Laughren

**Affiliations:** 1 Department of Economics, Simon Fraser University, Burnaby, Canada; 2 Smith School of Business, Queen’s University, Kingston, Canada

**Keywords:** Task completion, Present bias, Time inconsistency, Procrastination, D90

## Abstract

We conduct an experiment where participants make choices between completing a task now or waiting to complete it in the future. We vary the dates when a task can be completed and the effort required at each date. We infer participants’ preferences for when to complete a task and their expectations about how their future preferences will differ from their current ones. Our findings indicate that most participants prefer to complete tasks immediately, even if it demands more effort than waiting. Their choices generally align with the principles of time consistency, monotonicity, and time invariance. We show that quasi-hyperbolic discounting, anticipatory utility, fixed costs, decision costs, and cost-of-keeping-track are all unable to provide a reasonable account of both our findings and related experiments.

**Supplementary Information:**

The online version contains supplementary material available at 10.1007/s10683-024-09824-2.

Many economic decisions involve a trade-off between benefits and costs in the present and in the future: how much to consume versus save for later, whether to exercise or not, and whether to complete an onerous task today or to postpone it. These decisions involve individuals making choices at multiple points in time with no ability to commit to future choices. Until recently, most intertemporal choice experiments only studied choices over delayed monetary rewards made at a single point in time (e.g. Coller & Williams 1999; Harrison et al. 2002; Andreoni & Sprenger 2012). As a result, economists have limited experimental evidence on time inconsistency for non-monetary rewards and even less evidence on how people form expectations about their own future time inconsistency. We contribute by studying participants’ decisions to complete a task or delay in an environment without commitment in order to reveal their sophistication about their own time inconsistency.

We introduce a multi-day experimental design to observe task completion decisions over real effort and time. Each participant must complete a real effort task that consists of a number of chores to be eligible for a fixed payment at the end of the week. Crucially, a participant’s initial choices cannot commit their future choices except by completing the task. Each participant is presented with multiple two-date and three-date *effort schedules* that specify the number of chores associated with each of the available dates. For each effort schedule that includes the current day, the participant must indicate their choice to either do the task “today” or “not today”. If they choose “today”, they must complete the specified number of real effort chores by the end of today to be eligible for payment. If they choose “not today”, then the next day they face all effort schedules for which they previously selected “not today” plus those schedules for which “today” was not previously available. Across schedules we vary both the effort required on each available day and the days available to complete the task. In each two-date schedule, each decision at the earlier date elicits a preference at the earlier date. In each three-date schedule, each decision at the earlier date reflects both preferences and expectations about future behavior. Combining observations from two- and three-date effort schedules allows us to test axioms about intertemporal preferences and expectations about future preferences using choice data.

Our experiment is designed to test three normative axioms of intertemporal choice: sophistication, time consistency and time invariance. The *sophistication* axiom requires that a person correctly forecasts their future choices. The *time consistency* axiom requires that if a person chooses an option over another today, they would wish to make that same choice between options tomorrow if the consequences of the two actions have not changed. The *time invariance* axiom requires that if a person chooses one option over another today, they would make the same choice tomorrow if the consequences of each action were shifted one day in the future (Halevy, 2015). Each of the three axioms restricts the relationship between choices at two points in time, and there is limited body of experimental work that tests them using choices. Our design uses combinations of two- and three-date effort schedules that allow us to test each axiom for each experiment participant.

We find that participants demonstrate a tendency to complete a task immediately, even when delaying would have reduced the number of required chores. Specifically, 78% of two-date choices are resolved in favor of completing the task immediately, including 52% of two-date choices in which delaying reduces the number of chores. In two-date choices a participant’s beliefs about their future behavior are trivial and thus the immediate completion tendency we document for real effort tasks does not arise from sophistication about inconsistent preferences. Participants exhibit a high degree of time consistency despite the considerable power of our experiment to detect violations of this axiom. We find that 50 of 82 participants are time consistent in every test, and 29 of these 50 always choose to complete the task on Day 1 when it is available regardless of the effort trade-off.

We discuss how our findings relate to several theories of intertemporal choice. A structural model of quasi-hyperbolic discounting requires a future bias ($\beta >1$) to capture the early completion tendency we observed, which contradicts the intuition that people prefer to delay unpleasant tasks (e.g. O’Donoghue & Rabin 1999). Our design controls for fixed costs with a daily login requirement, equalizes costs of keeping track by using reminders, and we show that decision costs cannot rationalize our findings. A model of anticipatory utility like that of Loewenstein (1987) can generate an early completion tendency, but we show that the assumptions required would incorrectly predict an early completion tendency in the convex time budget (CTB) experiments of Augenblick et al. (2015). A model in which a person discounts future goods and bads by a subjective and fixed amount, i.e., a subjective fixed cost of delay (Hardisty et al., 2013) can generate present bias for goods and an immediate completion tendency for bads like real effort tasks (though our design controls for actual fixed costs). An alternative explanation is that people are biased to get tasks started, as seen in some psychology experiments following Rosenbaum et al. (2014). Our results suggest that intertemporal choices are affected by a factor distinct from present bias that is amenable to behavioral modeling.

**Related Literature** There is an extentsive experimental literature on intertemporal choice that studies preferences over delayed monetary rewards revealed at one point in time (e.g. Coller & Williams 1999; Harrison et al. 2002). Since money can be saved and borrowed such experiments should not, in principle, reveal intertemporal preferences if participants broadly bracket their experimental choices with opportunities outside of the lab (Cubitt & Read, 2007; Cohen et al., 2020). Thus, some intertemporal choice experiments use less-fungible rewards that will be consumed immediately like snacks (Read et al., 1998)) and real effort tasks (Augenblick et al., 2015; Carvalho et al., 2016; Augenblick, 2018; Augenblick & Rabin, 2019; Le Yaouanq & Schwardmann, 2019; Bisin & Hyndman, 2020; Breig et al., 2020; Hardisty & Weber, 2020; Fedyk, 2021; Zou, 2021)). Most papers in this literature find that subjects are present biased on average, a finding less pronounced for monetary rewards (Augenblick et al., 2015); see also meta studies by Imai et al. (2020) and Cheung et al. (2021)). Our design uses a real effort task from Augenblick et al. (2015).

Several papers in this literature indirectly test for a person’s sophistication or naivete about their own time inconsistency by measuring demand for commitment devices (e.g. Ashraf et al. 2006; Augenblick et al. 2015; see a review in Bryan et al. (2010)) or by comparing elicited beliefs to actual future choices (Augenblick & Rabin, 2019; Hardisty & Weber, 2020)). In contrast, our design elicits choices at different points in time in an environment where a participant cannot commit their future choices. This allows us to employ Freeman’s (2021) approach to test both sophistication and naivete about time inconsistency for each participant. Our design is motivated by a literature that commonly finds evidence of time inconsistency and that has found widely different degrees of sophistication about it (Ashraf et al., 2006; Augenblick et al., 2015; Augenblick & Rabin, 2019)). Yet, unlike this literature, we find little evidence of time inconsistency and thus have little to say about sophistication and naivete.

Most of the earlier literature on intertemporal choice only studies choices made at one point in time and thus cannot directly test time consistency or time invariance, with some exceptions,^1^ For instance, Read et al. (1998) have their participants choose a post-lunch snack both a week in advance and again the day they consume the snacks. They find participants choose unhealthy snacks more frequently when choosing same-day than in-advance. Augenblick et al. (2015) study participants’ allocations of real effort chores between earlier and later dates, both when the earlier date is in the future and when it is immediate. Similarly, Augenblick and Rabin (2019) elicit participant preferences over quantities of delayed real effort chores at different points in time, and separately elicit participant beliefs about their future preferences. These two real effort experiments both find that participants tend to prefer to delay effortful chores, and exhibit present bias by exhibiting a disproportionate preference to delay immediate effort. In the domain of monetary rewards, Halevy (2015) studies a design in which participants report their preferences between smaller-sooner versus larger-later monetary payments in successive weeks to test time invariance and time consistency. Halevy finds that over half of participants are time consistent and roughly half of all participants satisfy time invariance. Compared to this literature, our experiment is primarily designed to test sophistication without eliciting beliefs or explicitly measuring demand for commitment, and secondarily to test time consistency and time invariance in a real effort design.

## Theoretical framework

We study how a person’s decisions to complete a one-time task are affected by the options they have to complete the task in the future. Consider a person who must complete a real effort task exactly once. When first confronted with the task, the person is informed of the two or three different days on which they can do the task and how much effort they must exert to complete it on each of those days. On each day before the last day (deadline) the person can either complete the task or delay completion to a later date, but they cannot commit their future behavior except by completing the task.

Each *effort schedule* can be represented as a vector of effort requirements, one for each possible date. We write $\left( e_{1},e_{2},e_{3},\emptyset \right)$ to denote the effort schedule in which $e_{t}$ is the effort required to complete the task on Day *t* and the task cannot be completed at $t=4$. We consider three-date effort schedules with three consecutive completion options of the form $\left( e_{1},e_{2},e_{3},\emptyset \right)$ and $\left( \emptyset ,e_{2},e_{3},e_{4}\right)$, as well as two-date effort schedules that are derived by removing one option (i.e. changing an $e_{t}$ to $\emptyset$). Each statement below about effort schedules derived from $\left( e_{1},e_{2},e_{3},\emptyset \right)$ applies to analogous statements about effort schedules derived from $\left( \emptyset ,e_{2}^{\prime },e_{3}^{\prime },e_{4}^{\prime }\right)$ by shifting all dates forward by one, i.e., when $e_{1}=e_{2}^{\prime }$, $e_{2}=e_{3}^{\prime }$, and $e_{3}=e_{4}^{\prime }$.

Let *c* denote a *completion function* that returns the time, from among those available, at which the person would complete the task given an effort schedule. That is, $t=c\left( e_{1},e_{2},e_{3},\emptyset \right)$ denotes that the person would complete the task at time *t* if they faced $\left( e_{1},e_{2},e_{3},\emptyset \right)$, where *t* must be either 1, 2, or 3 in this case.^2^

To identify time inconsistency and distinguish sophistication from naivete, we assume that we observe a participant’s completion function over quadruples of related effort schedules of the form $\left( e_{1},e_{2},e_{3},\emptyset \right) ,$ $\left( e_{1},e_{2},\emptyset ,\emptyset \right)$, $\left( e_{1},\emptyset ,e_{3},\emptyset \right)$, and $\left( \emptyset ,e_{2},e_{3},\emptyset \right)$. We refer to such a quadruple of effort schedules as a *quad*. In this setting, a completion function is *time consistent *within a quad if it exhibits no choice cycles over two-date effort schedules: (i) if $2=c\left( e_{1},e_{2},\emptyset ,\emptyset \right)$ and $1=c\left( e_{1},\emptyset ,e_{3},\emptyset \right)$, then $2=c\left( \emptyset ,e_{2},e_{3},\emptyset \right)$, and (ii) if $1=c\left( e_{1},e_{2},\emptyset ,\emptyset \right)$ and $3=c\left( e_{1},\emptyset ,e_{3},\emptyset \right)$, then $3=c\left( \emptyset ,e_{2},e_{3},\emptyset \right)$.^3^

When a person faces a three-date effort schedule, they face not only a trade-off between their desire to not exert effort today and their desire to avoid effort later, but must also forecast their future choices to assess how to make this trade-off because they cannot commit their future behavior. Freeman (2021) shows that if a person is time inconsistent, observing a choice reversal can reveal their sophistication or naivete about their time inconsistency.

We define doing-it-later reversals and show how they reveal naivete. A completion function exhibits a *doing-it-later* *reversal *within a quad if (i) $3=c\left( e_{1},e_{2},e_{3},\emptyset \right)$ and $1=c\left( e_{1},\emptyset ,e_{3},\emptyset \right)$, or (ii) $2=c\left( e_{1},e_{2},e_{3},\emptyset \right)$ and $1=c\left( e_{1},e_{2},\emptyset ,\emptyset \right)$. To see why reversal (i) reveals naivete, notice that if a person would do it at $t=1$ when facing $\left( e_{1},\emptyset ,e_{3},\emptyset \right)$, they reveal that their $t=1$ preference to complete the task at $t=1$ over waiting until $t=3$. Since they cannot commit, they would only initially delay when facing $\left( e_{1},e_{2},e_{3},\emptyset \right)$ and then complete the task at $t=3$ if they incorrectly (i.e. naively) believe that they will complete it at $t=2$. This illustrates how a doing-it-later reversal reveals naivete.

We next define doing-it-earlier reversals and show how they reveal sophisitication. A completion function exhibits a *doing-it-earlier* *reversal *within a quad if $1=c\left( e_{1},e_{2},e_{3},\emptyset \right)$ and either (i) $2=c\left( e_{1},e_{2},\emptyset ,\emptyset \right)$ or (ii) $3=c\left( e_{1},\emptyset ,e_{3},\emptyset \right)$. To see why reversal (i) reveals sophistication, notice that if a person would complete the task at $t=2$ when facing $\left( e_{1},e_{2},\emptyset ,\emptyset \right)$, they reveal their $t=1$ preference to wait to do it at $t=2$ over completing it at $t=1$. This person would only complete the task at $t=1$ when facing $\left( e_{1},e_{2},e_{3},\emptyset \right)$ if they believe that their $t=2$ choice will be to complete it at the currently-less-preferred time $t=3$. In this case, completing the task at $t=1$ reveals that the person anticipates their own inconsistency between their $t=1$ and $t=2$ preferences and responds to it. This illustrates how a doing-it-earlier reversal reveals sophistication.

Some completion functions are neither time consistent within a quad nor do they exhibit a reversal within a quad. A completion function is* non-Strotzian* within a quad if it cannot be rationalized by *any* $t=1$ utility function over when to complete the task. Non-Strotzian completion functions can be divided into those in which the person initially chooses “not today” in $\left( e_{1},e_{2},e_{3},\emptyset \right)$, suggestive of a preference for flexibility, versus those in which the person chooses “today” in $\left( e_{1},e_{2},e_{3},\emptyset \right)$, suggestive of a preference for commitment.^4^

To illustrate how we use these definitions to analyze choices, Table 1 shows an example classification of four different completion functions (named Diego, Dillon, Norah, and Tim) within the choice quad derived from effort schedule $(16,20,25,\emptyset )$.

We also test two common assumptions about completion functions, monotonicity and time invariance, that restrict choice across quads. *Monotonicity* requires that if $e_{1}^{\prime }\le e_{1}$, $e_{2}^{\prime }\ge e_{2}$, and $1=c\left( e_{1},e_{2},\emptyset ,\emptyset \right)$, then $1=c\left( e_{1}^{\prime },e_{2}^{\prime },\emptyset ,\emptyset \right)$, with analogous requirements for all comparable two-date effort schedules. *Time invariance* requires that if an effort schedule is identical to another except that all effort requirements are shifted by one day, then the completion time also shifts by one day. For example, if $e_{1}=e_{2}^{\prime }$ and $e_{2}=e_{3}^{\prime }$, then time invariance requires that $1=c\left( e_{1},e_{2},\emptyset ,\emptyset \right)$ if and only if $2=c\left( \emptyset ,e_{2}^{\prime },e_{3}^{\prime },\emptyset \right)$.

**Table 1:** Classification of four completion functions within one quad

Chore Requirements if Completed on				Work Day Observed			
Day 1	Day 2	Day 3	Day 4	Diego	Dillon	Norah	Tim
16	20	$\emptyset$	$\emptyset$	2	2	1	1
16	$\emptyset$	25	$\emptyset$	1	1	1	1
$\emptyset$	20	25	$\emptyset$	3	3	3	3
16	20	25	$\emptyset$	1	3	3	1
Analysis							
Diego	$1=c(16,20,25,\emptyset )$ and $2=c(16,20,\emptyset ,\emptyset )$ is a doing-it-earlier reversal						
Dillon	$3=c(16,20,25,\emptyset )$ and $1=c(16,\emptyset ,25,\emptyset )$ is a doing-it-later reversal						
Norah	$1=c(16,20,\emptyset ,\emptyset )=c(16,\emptyset ,25,\emptyset )\ne c(16,20,25,\emptyset )$ is non-Strotzian						
Tim	$1=c(16,20,\emptyset ,\emptyset )=c(16,\emptyset ,25,\emptyset )=c(16,20,25,\emptyset )$ is time consistent						

## Experimental design

We design a real effort experiment to obtain data on participants’ task completion decisions and test sophistication, time consistency, and time invariance. Our four-day experiment presents each participant with two- and three-date effort schedules. Each participant makes “today” or “not today” decisions from effort schedules, which provide us with data on their completion function for each effort schedule. We selected effort schedules organized in quads to test time consistency and to use reversals to identify sophistication and naivete.

To observe a participant’s decisions in multiple different effort schedules we employ the random incentive system, providing incentive for a participant to report their true preferences of whether to work or not on each day. On the first day of the experiment, the participant is presented with all effort schedules for the experiment and is informed that one of these has been randomly chosen and will be implemented: the *schedule-that-counts*. The participant then must choose to complete chores “today” or “not today” for every effort schedule in which a $t=1$ option is available. If they choose “today” in the schedule-that-counts, then they complete the required number of extra chores today. Otherwise, when they log in the next day, they face a “today” or “not today” decision for those effort schedules with a non-trivial choice unless they previously chose “today” for that schedule.^5^ This provides each participant with the incentive to make each decision as if it were the schedule-that-counts while allowing us to observe decisions from many effort schedules.

Our experiment presents each participant with effort schedules that are part of quads with completion options on either $t=1,2,3$ or $t=2,3,4$. We construct *effort profiles* that specify the number of chores required to complete the task on each of the three consecutive available days. We selected effort profiles to be able to detect varying degrees of present and future bias; Table 3 displays the effort profiles we use. For each effort profile, the experiment includes quads of effort schedules with completion options on $t=1,2,3$ and $t=2,3,4$.The latter effort schedules are obtained by shifting the former by one day, which enables us to test time invariance. We thus observe each participant make up to eight choices for one effort profile: one quad with opportunities to work on $t=1,2,3$ and one quad with options on $t=2,3,4$. Some choices are censored when participants complete their extra chores early in their schedule-that-counts, but this design combined with the random incentive system ensures that each participant has at least a $\frac{5}{8}$ chance of making choices at $t=2$.

There are 24 possible combination of choices that a participant can make within a quad. We categorize each combination of choices as time consistent, a doing-it-earlier reversal, a doing-it-later reversal, non-Strotzian, or censored, as illustrated in Table 2. Seven of the 24 possible choice combinations can be rationalized by a time consistent completion function. Six possible choice combinations contain a doing-it-earlier reversal and two contain a doing-it-later reversal. These choice combinations are consistent with maximizing preferences in each period combined with, respectively, sophisticated and naive beliefs about future preferences. Five possible choice combinations are neither time consistent nor exhibit a reversal within a quad; we categorize these choice combinations as non-Strotzian. We further divide these into those in which the participant initially delays in $\left( e_{1},e_{2},e_{3},\emptyset \right)$, suggestive of a preference for flexibility, versus those in which the participant completes it immediately when facing $\left( e_{1},e_{2},e_{3},\emptyset \right)$, suggestive of a preference for commitment. We classify as *censored *four choice combinations in which the participant chooses to delay for $\left( e_{1},e_{2},e_{3},\emptyset \right)$ and we do not observe $t=2$ choices, as censoring precludes a useful classification of such choices.

**Table 2:** Identification of all observable choice combinations within a quad

$c\left( e_{1},e_{2},\emptyset ,\emptyset \right)$	$c\left( e_{1},\emptyset ,e_{3},\emptyset \right)$	$c\left( \emptyset ,e_{2},e_{3},\emptyset \right)$	$c\left( e_{1},e_{2},e_{3},\emptyset \right)$	Choice Classification	Preference
1	1	$\emptyset$	1	Time Consistent	t = 1
1	1	2	1		t = 1
1	1	3	1		t = 1
1	3	3	3		t = 3
2	1	2	2		t = 2
2	3	2	2		t = 2
2	3	3	3		t = 3
1	3	$\emptyset$	1	Reversal	Earlier
1	3	2	1		Earlier
1	3	3	1		Earlier
2	1	$\emptyset$	1		Earlier
2	1	2	1		Earlier
2	1	3	1		Earlier
1	3	2	2		Later
2	1	3	3		Later
1	1	2	2	Non-Strotzian	Flexibility
1	1	3	3		Flexibility
2	3	$\emptyset$	1		Commitment
2	3	2	1		Commitment
2	3	3	1		Commitment
1	1	$\emptyset$	$\emptyset$	Censored	Censored
1	3	$\emptyset$	$\emptyset$		Censored
2	1	$\emptyset$	$\emptyset$		Censored
2	3	$\emptyset$	$\emptyset$		Censored

The above table extends to quads derived from $\left( \emptyset ,e_{2},e_{3},e_{4}\right)$ by adding 1 to every integer in the table, shifting all efforts and $\emptyset$s in the header one position to the right while adding a $\emptyset$ in the first position of each effort schedule

**Implementation** We recruited 101 participants from the Simon Fraser University Experimental Economics Laboratory Research Participation System. Experiments were conducted entirely online. After signing up, each participant attended a live introductory Zoom session on a Friday. At the introductory session, an experimenter collected a consent form, read instructions aloud, and gave participants the opportunity to ask questions through a confidential chat. After answering all questions, participants were asked to demonstrate that they were able to sign-in to the online experiment interface using the university’s secure sign-in and complete one chore.^6^ The experimenter provided technical support until all participants were successful. Each participant was paid $7 (CAD) for participating in the introductory session.

The experiment then took place the following Monday to Thursday. To complete the experiment, a participant was required to login to the experimental web interface on each of Monday, Tuesday, Wednesday, and Thursday. Each participant was sent a reminder email on each morning with a link to the experiment. After login, each participant was required to complete one mandatory chore each day. If a participant had not already completed extra chores, they were required to make task-completion decisions for all effort schedules where they had not previously made a “today” choice and there were two or more completion dates remaining. If a participant chose “today” in the schedule-that-counts (or if they had previously chosen “not today” for that schedule and the only remaining date to complete extra chores is the current date) they were required to complete the specified number of chores for that schedule to complete the task before the end of the day (23:59 Vancouver time). Each participant received an all-or-nothing payment of $25 for completing all experiment requirements beyond the introductory session. All payments were made by email transfer on the Sunday following the final experiment deadline.

Out of 101 participants who attended the online introduction and received the participation payment, 89 started the experiment on Monday, of which 82 completed all requirements over the four days. A breakdown of the exact experiment stage at which each participant dropped out is available in Online Supplement B. The remaining analyses focus only on the 82 participants who completed all requirements.

The baseline number of chores (20) and length of chore (40 characters) were chosen so the session would require less than one hour of a participant’s time over the four days to complete all chores and make all decisions required for the $25 completion pay. Our chore is the same a Greek character transcription used by Augenblick et al. (2015); see Appendix Fig. 2 for a participant chore screen. A 40-character chore requires 40 button clicks with 100% accuracy. The median number of extra chores completed was 20.

**Table 3:** Experiment effort profiles

Effort	Effort	# Participants	# Quads	Versions
Over Time	Profile	Observed	Observed	
Increasing	14, 20, 28	45	76	V1, V2
	16, 20, 25	82	144	All
	18, 20, 22	82	144	All
	19, 20, 21	22	37	V2
Constant	20, 20, 20	82	144	All
Decreasing	22, 20, 18	37	68	V3
	25, 20, 16	37	68	V3
	Total	82	681	

Each effort profile describes the number of chores required if working on Day 1, Day 2, or Day 3 (or for working on Day 2, Day 3, or Day 4)

We ran three versions of the experiment, varying the effort profiles participants faced across versions. In the first two versions of the experiment we used quads designed to have power to detect a participant’s present bias and their sophistication or naivete about said bias. For our third version, we included quads designed to test whether some participants exhibit a negative discount rate by choosing to exert more effort and complete the task at an earlier date. Specifically, we conducted Version 1 in a single session with 23 participants starting on July 20, 2020. After observing many “today” choices in Version 1, we added the (19, 20, 21) effort profile to Version 2 to allow us to detect even small degrees of present bias. Version 2 was conducted in a single session with 22 participants starting on July 27, 2020. Still observing many “today” choices, we chose effort profiles for Version 3 that enable us to detect whether participants would work today if doing so increased the number of chores required, which would indicate an opposing preference to those generated by discounting and present bias. We conducted Version 3 in two sessions, with 15 participants starting March 8, 2021 and with 22 participants starting March 29, 2021.

Table 3 displays the effort profiles participants faced in each version of the experiment. The effort profiles listed in Table 3 were used to form two quads, one quad with the effort schedule having availability at $t=1,2,3$ and a second quad with the same effort schedule shifted one day to $t=2,3,4$.^7^

**Data Censoring** We do not always observe two full choice quads from each effort schedule because the day on which a participant completes their extra chores is endogenous. When a participant completes their payoff-relevant extra chores at $t=1$ (Monday), they make no further task completion decisions. In these cases, we obtain no data for $t=2,3,4$ effort schedules nor do we obtain $t=2$ decisions from $t=1,2,3$ effort schedules. This partial censorship also occurs for $t=2,3,4$ effort schedules when the extra chores are completed at $t=2$.

This endogenous censoring is inherent when studying any incentivized when-to-do-it choices. However, our design has a 1/2 probability that a $t=2,3,4$ schedule is the schedule-that-counts, and a 1/8 probability that a $t=1,2,3$ schedule with no option to do the extra chores on Monday is the schedule-that-counts. This design results in a 5/8 exogenous probability that a participant makes payoff relevant choices on at least two days. Our software randomly assigned 52 of 82 participants such an effort schedule, which we refer to as the* non-endogenous subsample*. We highlight this subsample when discussing results that otherwise could be subject to endogeneity. The remaining 30 participants generate data that is subject to endogenous censoring, including 5 participants who (endogenously) generate only censored quads.

**Statistical Power** We bootstrap likelihood-based confidence regions (Hall, 1987) over simulated data to estimate our statistical power when restricting attention to the 52 participants in our non-endogenous subsample. We simulate data and check whether the observed proportion of participants who are time consistent, naive, and sophisticated falls within a 95% confidence region of the null proportion. Ex-post power analysis treats our observed data as the null and the confidence region around the null covers less than 7% of the parameter space. So we have 93% power to reject an alternative observation that is drawn randomly from a uniform distribution over the parameter space. In Online Supplement C we provide additional details on the ex-post confidence region and a complementary ex-ante power analysis.

## Results

We designed our experiment to test sophisitication and naivete by observing choice reversals. However, we found a majority of participants displayed time consistency, primarily due to their tendency to complete the task as soon as possible. We begin by exploring this surprising result.

**RESULT 1: Participants’ choices show an immediate completion tendency. In two-date effort schedules where waiting requires the same or less effort, 65% of participant choices are to work “today”.**

Table 4 shows that over 75% of the individual choices from two-date effort schedules are choices to work “today”, including approximately half of two-date choices from the decreasing effort profiles (22, 20, 18) and (25, 20, 16).^8^ Given the median participant required 75 s per chore, this implies a willingness to exert around 6 extra minutes of effort to complete the extra chores early.

**Table 4:** Proportion choosing to work “today” in two-date effort schedules (Non-endogenous subsample)

Proportion choosing to work “today”					
Effort over time	Effort profile	$\left( e_{1},e_{2},\emptyset ,\emptyset \right)$	$\left( e_{1},\emptyset ,e_{3},\emptyset \right)$	$\left( \emptyset ,e_{2},e_{3},\emptyset \right)$	Total
Increasing	14, 20, 28	96%	88%	88%	90%
	16, 20, 25	90%	85%	90%	88%
	18, 20, 22	81%	88%	88%	86%
	19, 20, 21	92%	92%	83%	89%
Constant	20, 20, 20	77%	77%	85%	79%
Decreasing	22, 20, 18	57%	50%	61%	56%
	25, 20, 16	46%	43%	57%	49%
	TOTAL	77%	76%	81%	78%

65% of participant choices are to work “today” when combining constant and decreasing effort profiles

52% of participant choices are to work “today” when combining decreasing profiles

Next, we proceed to what we originally intended to be our main analysis by categorizing a participant’s choice quads into time consistent, doing-it-earlier reversal, doing-it-later reversal, or non-Strotzian, as shown inTable 2.

**RESULT 2: Overall, choices in 82% of uncensored quads are time consistent. At the individual level, 50 of 82 participants are time consistent in all of their uncensored quads.**

When all choice combinations over quads are considered regardless of censoring or endogeneity, 500 of 681 observations are time consistent. After removing the 83 censored observations, 498 of 598 (84%) uncensored choice combinations are time consistent, 397 of which exhibit a consistent preference to complete the extra chores on the first available day. Table 5 provides the classifications by effort profile and remarkably within every effort profile, over two-thirds (66%) of all participant choice quads are time consistent; the level or interest rate on effort does not appear to affect overall rates of time consistency.^9^ Among the time inconsistent choice combinations, we observe similar rates of reversals and non-Strotzian observations (9% and 6% of “TOTAL” observations in Table 5). If choice data were generated randomly by independently mixing at each choice (“RANDOM” in Table 5), 33% of choice combinations would be time consistent, and 35% would exhibit a reversal.

**Table 5:** Classifying choice combinations within a quad by effort profile (all data)

Effort	Time Consistent			Reversal		Non-	Censored	Quads
Profile	1st day	2nd day	3rd day	earlier	later	Strotz		Observed
14, 20, 28	72%	3%	7%	4%	3%	7%	5%	76
16, 20, 25	69%	2%	6%	8%	1%	3%	10%	144
18, 20, 22	65%	4%	5%	8%	1%	7%	11%	144
19, 20, 21	65%	5%	5%	5%	3%	5%	11%	37
20, 20, 20	51%	6%	8%	10%	1%	7%	16%	144
22, 20, 18	37%	7%	24%	7%	0%	9%	16%	68
25, 20, 16	38%	7%	26%	10%	0%	3%	15%	68
TOTAL	58%	5%	10%	8%	1%	6%	12%	681
uncensored	66%	5%	12%	9%	1%	2%		598
RANDOM	13%	10%	10%	25%	10%	23%	9%	
uncensored	14%	11%	11%	28%	11%	25%		

RANDOM is the expected proportion if all choices are random and independent

“1st day” is $t=1$ in $(e_{1},e_{2},e_{3},\emptyset )$ effort schedules and $t=2$ in $(\emptyset ,e_{2},e_{3},e_{4})$ effort schedules

**Table 6:** Classifying choice combinations within a quad by effort profile (non-endogenous subsample)

Effort	Time Consistent			Reversal		Non-	Censored	Quads
Profile	1st day	2nd day	3rd day	earlier	later	Strotz		Observed
14, 20, 28	79%	4%	0%	4%	4%	8%	0%	24
16, 20, 25	75%	2%	6%	12%	0%	6%	0%	52
18, 20, 22	73%	4%	4%	10%	2%	8%	0%	52
19, 20, 21	75%	0%	0%	8%	8%	8%	0%	12
20, 20, 20	60%	10%	2%	12%	4%	13%	0%	52
22, 20, 18	36%	14%	29%	4%	0%	18%	0%	28
25, 20, 16	32%	14%	36%	14%	0%	4%	0%	28
TOTAL	63%	7%	10%	10%	2%	9%		248

This table only uses only the $(e_{1},e_{2},e_{3},\emptyset )$ effort schedules, and only the 52 participants whose randomly assigned schedule-that-counts does not include $e_{1}$

The full set of data in Table 5 are subject to endogenous sampling and censoring. Table 6 displays results for the non-endogenous subsample, and further drops the choice combinations over quads derived from $(\emptyset ,e_{2},e_{3},e_{4})$ effort schedules since they are subject to endogenous observation of choices at $t=3$ (Wednesday). The data in Table 6 have zero censored observations by construction, yet still exhibit a very similar mix of choice combinations to the “Total uncensored” data from Table 5.

Within the non-endogenous subsample, 28 of 52 participants are time consistent in 100% of observed choice combinations, and 18 of these 28 chose to work “today” in every choice. Since we observe this subsample make choices on at least two days, all of these tests of time consistency are non-trivial. All remaining tables in the main text of results include only this non-endogenous subsample – though the similar values in Tables 5 and 6 suggest that data censoring does not appear to drive our results on time consistency.

The remaining 30 of 82 participants were randomly assigned a schedule-that-counts which allowed them to complete their extra chores on Monday, and this subsample is subject to endogenous selection. The participants in this set who chose to work “today” for their schedule-that-counts do not make any more decisions after Monday.^10^ We find that 17 of these participants exclusively generate choice combinations that are time consistent, and another 5 only generate censored choice combinations, and thus satisfy time consistency trivially. The remaining 8 of these participants generated at least one reversal or non-Strotzian choice combination.

Time consistency is tested using a choice combination over a single quad, but an additional consideration is whether a participant’s choices are collectively sensible when looking across quads.

**RESULT 3: Overall, fewer than 5% of observations need to be dropped to make every participant consistent with monotonicity. Among the participants who are time consistent** ***within *every quad,** **90% also demonstrate monotonicity** ***across* all quads.**

Monotonicity links preferences across effort values and requires participants to consistently prefer exerting less effort while controlling for completion time. We evaluate whether a participant violates monotonicity, considering every two-date choice across all quads in the experiment.

We count the total number of monotonicity violations for each participant. We find that 58 of 82 participants (71%) demonstrate no violations of monotonicity in their choice data. Of the 50 participants who were time consistent in 100% of their classified choice combinations, only 5 made a choice violating monotonicity, thus 45 of 82 participants were both time consistent and monotonic in all choices.

**Fig. 1:**
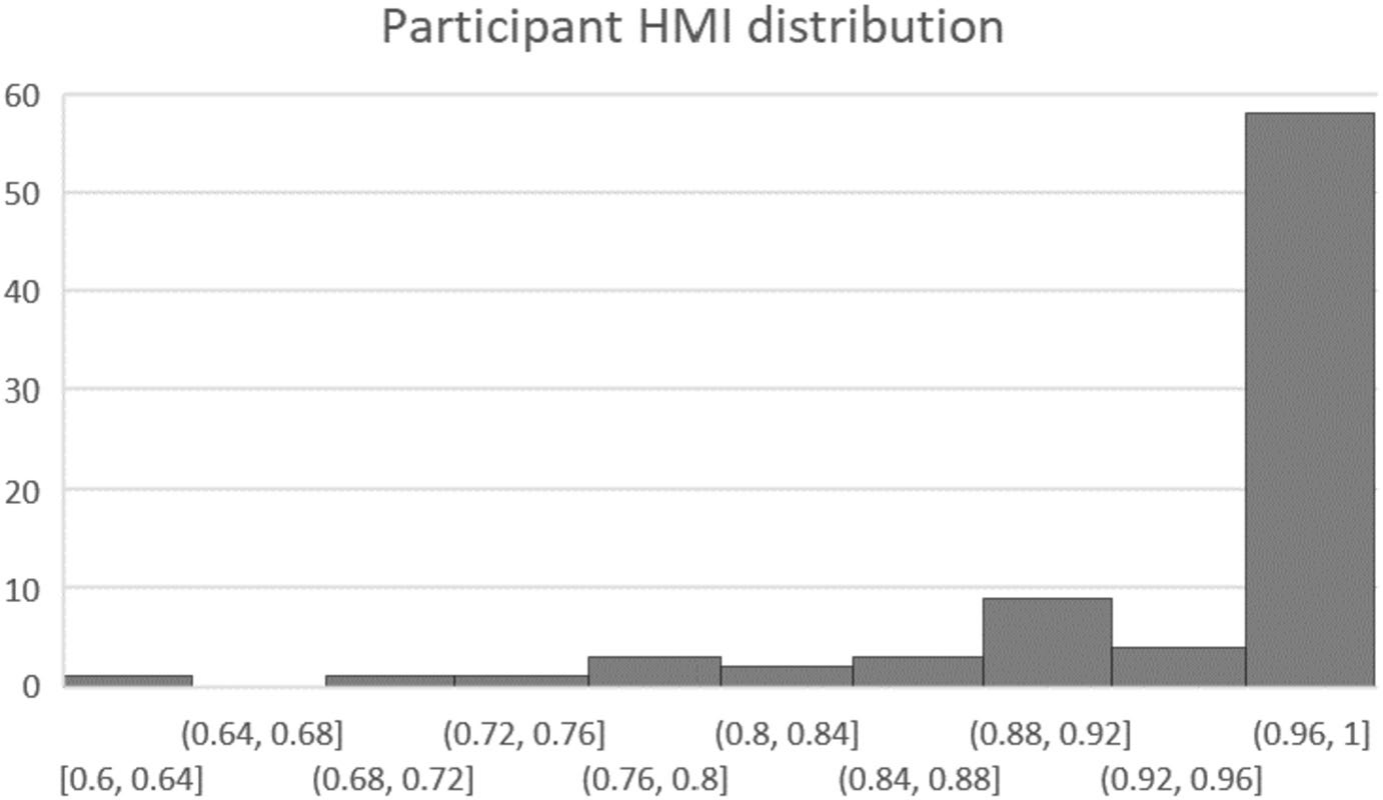
Participant Houtman-Maks index - monotonicity

For those participants who do violate monotonicity, we use the Houtman-Maks Index (HMI) to represent the maximal proportion of data which can be collectively consistent with monotonicity (Houtman and Maks, 1985; Heufer and Hjertstrand, 2015; Demuynck and Hjertstrand, 2019). This involves a simple linear optimization for each participant, minimizing the number of observations removed, subject to the constraint that there are zero monotonicity violations in the remaining dataset. In total, 76 of 1726 observations are removed for a weighted mean HMI of 0.955, and the mean HMI among those with at least one violation is 0.86. The distribution of HMI by participant in Fig. 1 further demonstrates that monotonicity violations are rare and concentrated in a minority of individuals.

When the same effort tradeoff is observed on different days, a participant who makes a different “today” or “not today” choice has violated time invariance. A violation of time invariance could suggest an unobserved preference to complete the task on a specific day.

**RESULT 4: Time invariance is satisfied in 79% of comparable decision pairs.**

Time invariance requires us to compare a participant’s choices in $t=1,2,3$ quads to their analogous choices in $t=2,3,4$ quads. Restricting attention to binary choices from the non-endogenous subsample, there are 496 total possible tests of time invariance.^11^ Time invariance is satisfied in 79% of tests, and 23 of 52 participants in the non-endogenous subsample satisfy time invariance in every test.

Table 7 displays the proportion of two-date choices that violate time invariance when we observe a participant make choices from comparable effort schedules on two different days. The relative scarcity of violations of time invariance suggest specific day-of-the-week preferences are driving choices in at most 22% of tests. The number of chores and the interest rate on effort do not appear to systematically affect the rate of failure of time invariance across schedules (Appendix Table 10).

**Table 7:** Time invariance violations by choice set (non-endogenous subsample)

1st Day Choice, 2nd Day Choice			
Effort schedule type	Time invariant	“today”, “not today”	“not today”, “today”
$\left( e_{1},e_{2},\emptyset ,\emptyset \right)$	79%	8%	13%
$\left( e_{1},\emptyset ,e_{3},\emptyset \right)$	78%	8%	13%
TOTAL	79%	8%	13%

The small response to a negative interest rate on effort apparent in Table 6 indicates that choices are not well represented by a standard model of intertemporal preferences in which participants discount costly future relative to immediate effort. We conduct a structural estimation to facilitate a comparison of behavior in our experiment to existing work.

**RESULT 5: Structural estimation of a model of quasi-hyperbolic discounting yields** $\beta >1$, **capturing a strong tendency to complete real effort tasks immediately.**

We model the probability of choosing “today” as resulting from a latent utility model. Consider only the two-date decisions, and let $e_{t},e_{t+k}$ denote the effort requirements for periods *t* and $t+k$. Let $Y_{t}=1$ denote a “today” choice at *t* and $Y_{t}=0$ denote a “not today” choice. We assume that $Y_{t}=1\iff Y_{t}^{*}\ge 0$, where $Y_{t}^{*}$ represents the time-*t* (unobserved) utility difference between choosing “today” and “not today”. We specify a structural quasi-hyperbolic discounting model with a linear disutility-of-effort: $Y_{t}^{*}$= $U_{t}(Y_{t}=1,e_{t},e_{t+k})-U(Y_{t}=0,e_{t},e_{t+k})$ where $U(Y_{t}=1,e_{t},e_{t+k})=-\lambda e_{t}$ and $U(Y_{t}=0,e_{t},e_{t+k})=-\beta \delta ^{k}\lambda e_{t+k}$ with $\beta$ and $\delta$ scalar time preference parameters to be estimated.

The net utility of working “today” can be written as $Y_{t}^{*}=-\lambda e_{t}+\beta \delta \lambda e_{t+k}\mathbb {I}_{\{k=1\}}+\beta \delta ^{2}\lambda e_{t+k}\mathbb {I}_{\{k=2\}}$. We assume there is some variation in individual values of $Y_{t}^{*}$ due to individual preference shocks, and specify a logit regression $Y_{t}^{*}=x'b+\epsilon _{t}$, where $x'b=b_{0}e_{t}+b_{1}e_{t+k}\mathbb{I}_{\{k=1\}}+b_{2}e_{t+k}\mathbb{I}_{\{k=2\}}$ and $\epsilon_{t}\sim \Lambda (),$ a standard binary logit model with no intercept term.^12^ We recover estimates of $(b_{0},b_{1},b_{2})$ and use them to estimate $\hat{\beta }=-\frac{(b_{1})^{2}}{b_{0}b_{2}}$; $\hat{\delta }=\frac{b_{2}}{b_{1}}$; and $\hat{\lambda }=-b_{0}$.^13^ We cluster standard errors by participant, and recover asymptotic standard errors for the parameter estimates using the delta method. Parameter estimates and their asymptotic standard errors are presented in Table 8. We provide the underlying logit regression estimates of $(b_{0},b_{1},b_{2})$ and further details in Appendix Tables 11, 12, and 13.

**Table 8:** Results of structural logit estimation (non-endogenous subsample)

Parameter	Estimate (Std. Error)	Confidence Intervals ($\alpha =0.05)$	
		Lower Bound	Upper Bound
Present Bias $\beta$	1.54	1.11	1.96
	(0.22)		
Discount Factor $\delta$	0.93	0.83	1.03
	(0.050)		
Disutility of Effort $\lambda$	0.14	0.06	0.22
	(0.042)		
Observations	743		
Clusters	52		

Estimated using binary effort schedules only. Standard errors of logit regression are clustered by individual participant. Asymptotic standard errors estimated using the delta method (derivation in Appendix)

Previous studies of intertemporal preference consistently estimate values of $\beta \le 1$, with the interpretation being that there is additional (non-geometric) discounting of all future periods relative to the present. Two recent meta analyses have found mean values of $\beta$ that are significantly less than one when conditioning on studies that used real-effort tasks (Imai et al., 2020) find an average $\beta$ of 0.88 across 9 studies) or non-monetary rewards (Cheung et al., 2021) find an average $\beta$ of 0.66 across 5 studies). Imai et al. (2020, Table 8) estimate that each of the main features of our experiment – non-monetary reward, conducted online, and ‘immediate: by end of day’ (as opposed to a longer time frame for immediate costs and rewards) – have a negative or insignificant effect on the value of $\beta$. Imai et al. (2020, p. 1804) demonstrate that the standard error of the $\beta$ estimate is negatively correlated with the value of the $\beta$ estimate in published real effort studies, “suggesting the existence of modest selective reporting in the direction of over-reporting $[\beta ]<1$ in studies using a real-effort task.”

The participants in this experiment had a clear disposition to complete the extra chores “today”, and this is reflected in the estimate of $\beta >1$, as caring more about future utility than today’s utility would result in the observed participant disposition to complete the task today (with 580 of 743 two-date choices to work “today”). The estimated value of the disutility of effort parameter has the expected sign. Geometric discounting is identified from the difference in choices when the delay is $k=2$ days versus $k=1$, but does not appear to be a significant driver of choice since $\delta \approx 1$. However, the strong tendency to immediately complete the task immediately makes it difficult to precisely estimate $\beta$ and $\delta$ since any combination of $\beta \delta >1$ can drive such a tendency, and our estimates of both $\beta$ and $\delta$ are imprecise.

The difference between our results and the results from previous real effort experiments studying time preferences warrants further discussion. We next discuss six classes behavioral explanations for our findings.^14^ We compare the ability of each to fit both our data and the stylized findings of prior real effort experiments that found evidence of present bias in a CTB experiment (Augenblick et al., 2015).

## Discussion: explaining an immediate completion tendency

Our experiment is designed to measure a person’s sophistication or naivete about their own time inconsistency. Yet we discovered far more time consistency than we expected based on prior work from economics experiments (e.g. Thaler 1981; Read & Van Leeuwen 1998; Ashraf et al. 2006), including other experiments that use designs with similar real effort tasks (Augenblick et al., 2015; Augenblick & Rabin, 2019; Augenblick, 2018)). This appears to be driven by a strong tendency to complete tasks immediately – even when this requires additional effort. We estimate a structural model to compare our results to previous work and we find paramater values that imply our participants have future-biased preferences. While some previous studies using monetary or hypothetical rewards have found evidence of future bias (e.g. Sayman & Öncüler 2009; Attema et al. 2010; Takeuchi 2011; Montiel Olea & Strzalecki 2014; Aycinena et al. 2019, 2022), recent meta-analyses document that such a finding is the exception and not the norm, and has no published precedent in real effort tasks (Imai et al., 2020; Cheung et al., 2021).

Next we outline six decision models that can rationalize an early completion tendency and discuss their consistency with both our data and related experiments. For the purpose of this Discussion section, we take Augenblick et al. (2015) as a typical intertemporal choice experiment that uses real effort tasks and finds a preference to delay work and we evaluate alternative theoretical explanations against both our findings and theirs. Because we use their Greek transcription task and we both use student participants, there seem to be few economically-important differences in our designs that could explain why our participants seem to make qualitatively different trade-offs between earlier versus later effort. The one economically crucial difference between the designs is that our participants make decisions once-and-for-all, while Augenblick et al. participants make effort allocations at-the-margin: each of their decisions has a participant allocate required chores between an earlier and a later date along a continuous convex time budget (CTB).

**Quasi-hyperbolic discounting** We find quasi-hyperbolic discounting an unsatisfactory explanation for our findings. To rationalize the early completion tendency in our data with a structual model of quasi-hyperbolic discounting requires $\beta >1$, which corresponds to a future bias. In contrast, past applications are motivated by present bias ($\beta <1$, Laibson (1997), O’Donoghue and Rabin (1999)) and published experimental studies using real effort tasks have found evidence of present bias (Imai et al., 2020; Cheung et al., 2021). Our results thus present a puzzle relative to experiments that study intertemporal allocations involving real effort tasks that have been taken as evidence for present bias.^15^

One difference between our study and most existing literature that use real effort experiments to study intertemporal choice is that we use delays on the order of 1–2 days, whereas most existing work studies longer delays. However, Augenblick (2018) studies discounting in the same real effort task over delays as short as two hours and finds that two-thirds of discounting that occurs within one week occurs in the first day. We thus rule out our 1–2 day delay length as a possible explanation for the future bias we find.

**Anticipatory utility** Dread from anticipating the need to complete real effort tasks in the future can generate an immediate completion tendency, but could also lead to a bias to complete more chores earlier in CTB designs, making it unclear whether anticipatory utility is a satisfactory explanation for our results. In Online Supplement A, we modify Loewenstein’s (1987) model of anticipatory utility to allow for present bias and study its relation to our experimental design and CTB designs. We show that the parameter restrictions required to explain an immediate completion tendency in our experiment also implies future bias in a CTB design, the opposite of what Augenblick et al. (2015) and similar papers observe. This calls into question whether anticipatory utility is the right explanation for our findings.

More general models of anticipatory utility beyond Loewenstein’s may be more successful. Anticipatory utility will tend to lead people to postpone good things and speed up bad things – acting against standard discounting and present bias. But if loss aversion creates a stronger motive for real effort tasks than for receipt of good things, then anticipatory utility may be particularly relevant in our environment (Hardisty & Weber, 2020). However, this motive would apply in all real effort designs, not just ours. Thus it does not seem like an obvious approach to jointly explain our finding and present bias in CTB designs. One possibility is that anticipatory utility displays diminishing sensitivity to the quantity of effort in ways that are inconsistent with Loewenstein’s model. In the most extreme version of this, anticipatory utility would act like a fixed cost of having to complete the task in the future – like a a fixed cost of delay, which we discuss below.

**Decision costs** We rule out decision costs as an explanation for the early completion tendency we find. A participant who experiences a subjective cost of making each choice and fully integrates the random incentive scheme might be biased, relative to their underlying time preferences, to choose “today” in our experiment because this reduces their probability of having to make choices tomorrow and thereby avoids any future subjective decision costs. We find this explanation implausible in our setting for three reasons. First, our instructions and comprehension tests (while clear and complete) did not emphasize this relatively subtle aspect of the design. Second, a long experimental literature has tested whether people tend to make each choice in isolation or rationally account for the experiment’s incentive scheme – and this work has almost universally found that most people make each choice in isolation (Starmer & Sugden, 1991; Cubitt et al., 1998; Hey & Lee, 2005; Freeman & Mayraz, 2019). Third, this explanation should be more powerful on Monday than Tuesday: completing the task on Monday avoids 19 Tuesday choices (plus possibly avoids Wednesday choices), whereas completing the task on Tuesday avoids only 9 Wednesday choices. However, we see roughly the same degree of early completion bias in Monday and Tuesday choices: in Table 4 we see 77% early completion over $(e_{1},e_{2,},\emptyset ,\emptyset )$ schedules when 19 decisions could be avoided, but 81% early completion over $(\emptyset ,e_{2},e_{3},\emptyset )$ schedules when only 9 decisions could be avoided. For the effort profile (20, 20, 20), we see that early completion occurs in 77% of opportunities when 19 decisions could be avoided, but early completion occurs in 85% of cases for when only 9 decisions could be avoided. Our participants showed a stronger early completion tendency when the number of future decisions was lower, and this strongly suggests that a motive to avoid facing future decisions is not a good explanation of the early completion tendency we find.

**Cost of keeping track** Both our experiment and Augenblick et al. (2015) require subjects to log on and complete a minimal number of chores in all periods regardless of choices, and provide sign-in reminders to subjects. This makes an anticipation of a cost of keeping track Haushofer (2015) or of memory limitations Ericson (2017) not particularly compelling explanations for our finding.

**Fixed cost of delay** Hardisty et al. (2013) present a model in which a person makes intertemporal choices as if they experience a fixed cost of delay; a particular interpretation of this model might explain our findings, but we have some caveats. For receipt of goods, this model will result in behavior that looks like present bias. But for bads – like our real effort tasks – it could result in an immediate completion tendency over low stakes in spite of a preference for delay over high stakes.^16^

One possible weakness of this explanation is that both our design and Augenblick et al. (2015) require a login and mandatory chores in all periods. If accounted for by participants, all participants should experience both real and subjective fixed costs each period regardless of their choices. Thus any fixed costs of delay should not influence behavior in either of our experiments. However, Ellis and Freeman (2021) show that most people are well-described as narrow bracketers in a variety of domains. In our setting, a narrow bracketer only responds to the choice in front of them, and ignores the mandatory logins and chores when making each choice. We thus consider whether a fixed cost of delay combined with narrow bracketing can explain our results.

A person who experiences a subjective fixed cost of delay and brackets narrowly should exhibit an early completion tendency in our design. When subjective fixed costs are sufficiently large, they should also exhibit an early completion tendency in CTB designs whenever the stakes are sufficiently low for it to be worthwhile to complete all required chores immediately to avoid fixed costs. But conditional on making interior allocations in a CTB design fixed costs should not influence trade-offs, and thus if people are present biased after controlling for subjective fixed costs the CTB will only detect present bias and not subjective fixed costs.^17^ Thus, the combination of narrow bracketing and fixed cost discounting can perhaps accommodate both the early completion tendency we find and present-biased choices in the Augenblick et al. (2015) CTB design.

**Get-it-started bias** A bias to get tasks started can possibly explain our finding. Using a very different type of real effort task Rosenbaum et al. (2014) and Fournier et al. (2019a; b) document a bias to get tasks started. In our design, starting and finishing a task are tied, so a get-it-started bias would lead to an early completion tendency in our experiment. In a CTB, starting and finishing a task are de-coupled, so a participant can satisfy their get-it-started bias but still allocate effort to the future. If participants also exhibit present bias, a CTB design should detect this. Thus a get-it-started bias is potentially consistent with both our finding and the findings of Augenblick et al. (2015). We consider this the most plausible explanation for behavior in our experiment.

**Future research** Our findings present a challenge to the standard model of intertemporal choice, quasi-hyperbolic discounting, on a domain where previous literature suggests it ought to apply. Our findings also challenge existing models of anticipatory utility, although more general models of anticipatory utility might be more successful. We find a get-it-started bias to be a plausible though somewhat unsatisfying explanation, in part because it is far from standard models of intertemporal choice considered in the behavioral economics literature. Hardisty & Weber (2020, Experiment 3) find that participants are more prone to immediately eat bad-flavored jelly beans than good flavored jelly beans, and they link to anticipatory utility without providing any formal modeling. A carefully designed incentivized experiment could shed further light on why in some choices (like those in our experiment) participants exhibit an early completion tendency whereas in others they tend to delay. Further work is needed to understand whether more general models of anticipatory utility or discounting can provide a reasonable account of intertemporal decisions involving real-effort tasks.

We also explored two other standard properties assumed in most models of intertemporal choice—time invariance and monotonicity—and we do not detect systematic failures of either property. This suggests that these are both descriptively reasonable properties to retain.

## Supplementary Information

Below is the link to the electronic supplementary material.

Supplementary file 1 (pdf 894 KB)

## Figures and Tables

**Fig. 2:**
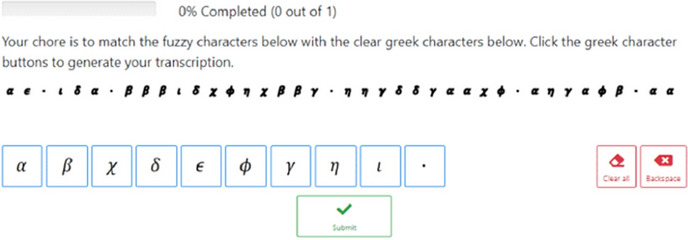
Experiment chore The status at the top of this figure implies this is the chore screen on a day when a participant has chosen to only complete the minimum of one chore

**Fig. 3:**
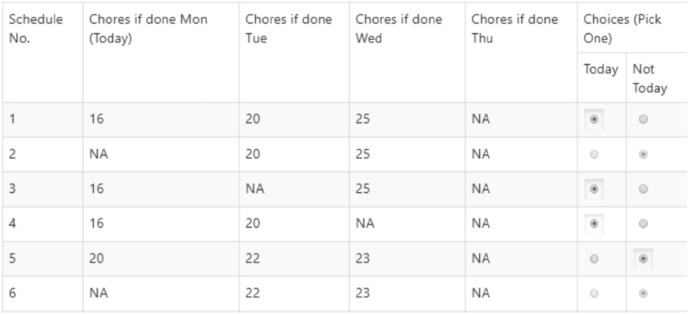
Excerpt of experiment decision table (Monday) Effort schedules like Schedule No. 2 and Schedule No. 6 are displayed to participants so they are aware of all possible schedules-that-count, but the buttons are fixed on “Not Today” for these schedules because they are Not Available (NA) to be completed on Monday

## Appendix

See Tables 9, 10, 11, 12, 13.

**Table 9:** Classifying choice combinations within a quad by effort profile (non-endougenous subsample)

Effort	Time Consistent			Reversal		Non-Strotz	Censored	Quads
Profile	1st day	2nd day	3rd day	earlier	later			Observed
14, 20, 28	83%	0%	0%	8%	4%	4%	0%	24
16, 20, 25	77%	2%	0%	8%	2%	2%	10%	52
18, 20, 22	73%	4%	2%	6%	0%	2%	13%	52
19, 20, 21	75%	8%	0%	0%	0%	0%	17%	12
20, 20, 20	65%	2%	6%	12%	0%	0%	15%	52
22, 20, 18	50%	0%	18%	11%	0%	4%	18%	28
25, 20, 16	57%	0%	18%	7%	0%	4%	14%	28
TOTAL	69%	2%	6%	8%	1%	2%	13%	248

This table only uses only the $(\emptyset ,e_{2},e_{3},e_{4})$ effort schedules, and only the 52 participants whose randomly assigned choice that counts does not include $e_{1}$

**Table 10:** Time invariance by effort schedule (non-endogenous subsample)

Effort Profile	First Day Choice, Second Day Choice		
	Time Invariant	“today”, “not today”	“not today”, “today”
14, 20, 28	90%	4%	6%
16, 20, 25	87%	7%	7%
18, 20, 22	80%	9%	12%
19, 20, 21	83%	8%	8%
20, 20, 20	70%	12%	17%
22, 20, 18	70%	11%	20%
25, 20, 16	75%	2%	23%
Grand Total	79%	8%	13%

**Table 11:** Structural logit estimates (non-endogenous subsample)

	*Dependent variable:*
	Today1
EffortToday	−0.140^***^
	(0.042)
EffortNotTodayK1	0.199^***^
	(0.041)
EffortNotTodayK2	0.185^***^
	(0.043)
Observations	743
Log Likelihood	−358.575
Akaike Inf. Crit	723.150

^*^p<0.1; ^**^p<0.05; ^***^p<0.01

**Table 12:** Variance-covariance matrix of structural logit estimates (Non-endogenous subsample)

	EffortToday	EffortNotTodayK1	EffortNotTodayK2
EffortToday	0.001803	$-0.001716$	$-0.001747$
EffortNotTodayK1	$-0.001716$	0.001716	0.001725
EffortNotTodayK2	$-0.001747$	0.001725	0.001826

**Table 13:** Variance-Covariance Matrix of Parameter Estimates using Delta Method

	CRow1	CRow2	CRow3
CRow1	0.046933	$-0.007522$	0.007851
CRow2	$-0.007522$	0.002545	$-0.000756$
CRow3	0.007851	$-0.000756$	0.001803

**Deriving parameter estimates and variance-covariance matrix for delta method**

We estimate the logistic regression:

$$y_{t}=b_{0}e_{t}+b_{1}\text {I}_{k=1}e_{t+k}+b_{2}\text {I}_{k=2}e_{t+k}+\epsilon _{t}$$

For our structural model, we are interested in the values of $\frac{b_{1}^{2}}{b_{0}b_{2}}$ $(=\beta$), $\frac{b_{2}}{b_{1}}$ ($=\delta$), and $b_{0}$ ($=\lambda$)

If $b\sim N(b^{\star },\Sigma )$ then the distribution of *f*(*b*) is $N(f(b^{\star }),C\Sigma C^{\prime })$ where $C=\nabla f(b)$.

Let $$f(b)=\left[ \begin{array}{c} \frac{b_{1}^{2}}{b_{0}b_{2}}\\ \frac{b_{2}}{b_{1}}\\ b_{0} \end{array}\right] $$. Then, $$C=\nabla f(b)=\left[ \begin{array}{ccc} -\frac{b_{1}^{2}}{b_{0}^{2}b_{2}} &{} \frac{2b_{1}}{b_{0}b_{2}} &{} -\frac{b_{1}^{2}}{b_{0}b_{2}^{2}}\\ 0 &{} -\frac{b_{2}}{b_{1}^{2}} &{} \frac{1}{b_{1}}\\ 1 &{} 0 &{} 0 \end{array}\right] $$.

The estimated variance matrix of interest is $C\hat{\Sigma }C^{\prime }$See Figs. 2, 3.

## Footnotes

1 Some earlier experiments observe decisions at multiple points in time (Carbone & Hey, 2001; Bone et al., 2009) including experiments that study dynamic choice under risk (Cubitt et al., 1998; Busemeyer et al., 2000; Hey & Lotito, 2009).

2 In our theoretical analysis that follows we assume that each person has, at each point in time, transitive preferences over completion time-effort pairs and a belief about their preference in the next period.

3 Notice that $c\left( e_{1},e_{2},\emptyset ,\emptyset \right)$ and $c\left( e_{1},\emptyset ,e_{3},\emptyset \right)$ reveal the the $t=1$ self’s preferences (which, assuming transitivity, can reveal the preference between completing at $t=2$ vs. $t=3$), whereas $c\left( \emptyset ,e_{2},e_{3},\emptyset \right)$ directly reveals the $t=2$ self’s preferences. Time consistency requires that the two selves’ preferences between doing it at $t=2$ vs. $t=3$ are the same.

4 Neither type of non-Strotzian completion function can be generated by a perception-perfect strategy (Freeman, 2021).

5 See Appendix Fig. 3 for a representative decision screen; complete instructions are available in Online Supplement E.

6 To help ensure the participants did not outsource decision making or task completion, on each day of the experiment participants were required to use the university secure sign-in with multi-factor authentication. We do not believe a participant is likely to give access to their university password and mobile phone in an attempt to outsource a $25 task.

7 A comprehensive list of effort schedules participants faced by version is provided in Online Supplement D.

8 This pattern is evident whether the first day is $t=1$ (as in Table 6) or is $t=2$ (as in Appendix Table 9), as both show over 60% of choices within a quad are time consistent with a preference for Day 1; this suggests the pattern is not simply a day of the week preference.

9 All participants made choices over effort profiles (16, 20, 25), (18, 20, 22), and (20, 20, 20) regardless of the experiment version they faced. There is no statistical difference in the rate of time consistency between these effort profiles. We fail to reject the null hypothesis that the rate of time consistency in effort profile (16, 20, 25) is drawn from the same distribution as the rate of time consistency in effort profile (20, 20, 20), (p = 0.09 using a Fisher exact test with n = 144 in each group total, or p = 0.31 with n = 130 and n = 120 uncensored group totals, respectively).

10 For example, suppose on Monday we observe “today” for $\left( e_{1},e_{2},\emptyset ,\emptyset \right)$, “not today” for $\left( e_{1},\emptyset ,e_{3},\emptyset \right)$, and “not today” for $\left( e_{1},e_{2},e_{3},\emptyset \right)$. If $\left( e_{1},e_{2},\emptyset ,\emptyset \right)$ is the schedule-that-counts, the participant completes extra chores Monday. Thus $c\left( e_{1},e_{2},e_{3},\emptyset \right)$ and $c\left( \emptyset ,e_{2},e_{3},\emptyset \right)$ are never observed, and the choice combination is censored. Table 2 shows the categorization of all observable choice combinations.

11 We conduct two tests per effort profile. We exclude the tripleton schedule comparison of $\left( e_{1},e_{2},e_{3},\emptyset \right)$ to $\left( \emptyset ,e_{2}^{\prime },e_{3}^{\prime },e_{4}^{\prime }\right)$ because a participant who chose “not today” at the first opportunity can have their $\left( e_{3}^{\prime },e_{4}^{\prime }\right)$ choice censored. We also exclude $\left( \emptyset ,e_{2},e_{3},\emptyset \right)$ to $\left( \emptyset ,\emptyset ,e_{3}^{\prime },e_{4}^{\prime }\right)$ choices because observations of the latter require observing choices on $t=3$ which is subject to endogenous selection.

12 Forcing the regression to an intercept at zero is equivalent to assuming that $Prob(Y_{t}=1|e_{t=0},e_{t+k}=0)=0.5$, which is true in this structural utility model.

13 We caution against overly interpreting our point estimates. The parameter $\lambda$ can be viewed as controlling sensitivity to effort or alternatively the degree of stochasticity, and our estimation cannot separate these interpretations. Similarly, notice that $-e_{t}>-\beta \delta ^{t+k}e_{t+k}$ if and only if $-e_{t}^{\gamma }>-\beta ^{\gamma }\delta ^{\gamma t+\gamma k}e_{t+k}^{\gamma }$ for every $\gamma >0$. Our design does not identify the curvature-of-disutility-of-effort parameter $\gamma$, so our point estimates of $\delta$ and $\beta$ cannot be directly compared to those in existing work that does attempt to identify $\gamma$ (e.g. Augenblick et al. 2015).However, if a person deterministically follows our model, it will accurately identify whether they exhibit present versus future bias even if it does not obtain the correct parameter. If the model is misspecified and people are heterogeneous, as we expect to be the case in our estimation and all other structural estimations of models of quasi-hyperbolic discounting, then our tests of $\beta \gtrless 1$ and $\delta \gtrless 1$ may have be invalid.

14 We thank the referees for constructive suggestions that helped us to better understand our findings.

15 Similar to our experiment, a low – or even negative – rate of discounting for bads has been documented in prior work on monetary discounting (e.g. Abdellaoui et al. 2013; see also Loewenstein and Prelec ’s 1992 discussion of gain-loss asymmetry), but not in real effort experiments.

16 Benhabib et al. (2010) propose a related model of fixed-cost discounting, however, their model predicts discounting and hence a desire to delay the receipt of bads, like our real effort tasks.

17 Only 31% of effort decisions involve a corner in (Augenblick et al., 2015) and only one person makes no interior allocations.
