# Supplementary material for: Task completion without commitment
Source: Exp Econ. 2024 May 14;27(2):273–98. doi: 10.1007/s10683-024-09824-2 (PMC11628449; doi:10.1007/s10683-024-09824-2)
Supplement: Supplementary file 1 — (pdf 894 KB) [file 10683_2024_9824_MOESM1_ESM.pdf]

Online Supplement A: Applying anticipatory utility to explain an immediate completion tendency

We take as our core anomaly that we find evidence for an immediate completion tendency in our all-or-nothing design, whereas prior work studying at-the-margin real-effort choices finds evidence of present bias – essentially the opposite finding.

We follow Loewenstein (1987) and model a person's time t evaluation of the desirability of completing e_{t+k} at $t+k$ according to a sum of the discounted disutility of effort and discounted disutility from anticipating future effort. We extend his model to allow for present biased discounting. Let $d : \mathbb{R}_+ \to \mathbb{R}_+$ denote a disutility-of-effort function, so that $d(e)$ gives the subjective cost of exerting e units of effort immediately. Let $\beta \in (0,1]$ denote a person's present bias, let $\delta \in (0,1]$ denote their standard discount factor. Thus, they assign subjective cost of $\beta\delta^k d(e)$ to the requirement to complete e units of effort $k \geq 1$ periods in the future. In addition to effort disutility, a person experiences disutility from anticipating future required effort. Let $\alpha \geq 0$ denote the weight they place on anticipation and let ρ denote their discount factor for anticipatory utility. We assume that the same present bias factor β also applies to anticipatory utility. At t, they thus experience disutility $-\alpha\beta\rho^k d(e_{t+k})$ of anticipating future effort e_{t+k} required in k periods. When $k > 1$, the person also expects that they will experience the negative anticipatory utility of $-\alpha\rho^{k-1} d(e_{t+k})$ at $t+1$, and they discount that future anticipatory utility according to $\beta\delta$. They analogously weight the future anticipatory utility they expect to experience at $t+2, \ldots, t+k-1$. Thus, at t the evaluates the overall disutility of having to complete e_{t+k} chores at $t+k$ as $-\beta\delta^k d(e_{t+k}) - \alpha\beta\sum_{\tau=0}^{k-1}\delta^\tau\rho^{k-\tau} d(e_{t+k})$.

We wish to derive parameter restrictions under which this model of anticipatory utility can rationalize our findings. We first calculate both the $t = 1$ self utility evaluations of three options: completing e_1 chores at $t = 1$, completing e_2 chores at $t = 2$, and completing e_3 chores at $t = 3$.

$t = 1$ evaluation:

$$
\begin{array}{llll}
t = 1 : & -\, d(e_1) & & \\
t = 2 : & -\, \alpha\beta\rho d(e_2) & -\beta\delta d(e_2) & \\
t = 3 : & -\, \alpha\beta\rho^2 d(e_3) & -\alpha\beta\delta\rho d(e_3) & -\beta\delta^2 d(e_3)
\end{array}
$$

Second, we calculate the $t = 1$ self's utility evaluations of the $t = 1$ and $t = 2$ options.

$t = 2$ evaluation:

$$
\begin{array}{lll}
t = 2 : & -\, d(e_2) & \\
t = 3 : & -\, \alpha\beta\rho d(e_3) & -\beta\delta d(e_3)
\end{array}
$$

When $\beta = 1$, discounting in the model is exponential, and anticipatory utility induces a time-inconsistent future bias. To illustrate with a simple numerical example, suppose $d(e) = e$, $\alpha = 1$, and $\delta = \rho = 1$. Then, at $t = 1$, the person would be indifferent between completing $e_1 = 24$ chores immediately, $e_2 = 12$ chores at $t = 2$, or $e_3 = 8$ chores at $t = 3$. At $t = 2$, they would be indifferent between $e_2 = 12$ chores at $t = 2$ and $e_3 = 6$ chores at $t = 3$.

To rationalize an immediate completion tendency in our finding, we need it to be the case that when $e_1 = e_2 = e_3$, the person would, at each earlier time, weakly prefer to do it now over delaying to either later option. This occurs when $1 \leq \min\{\alpha\beta\rho + \beta\delta, \alpha\beta\rho^2 + \alpha\beta\delta\rho + \beta\delta^2\}$.

We also wish to derive the parameter restrictions on this model that would rationalize findings that are taken as evidence of present bias in CTB designs like Augenblick et al. (2015). We first apply the model obtain the $t = 1$ self's evaluation of an allocation that requires e_2 chores at $t = 2$ and e_3 chores at $t = 3$.

$t = 1$ evaluation:

$$
-\, \alpha\beta\rho d(e_2) - \alpha\beta\rho^2 d(e_3) \qquad -\beta\delta d(e_2) - \alpha\beta\delta\rho d(e_3) \qquad -\beta\delta^2 d(e_3)
$$

Second,

$t = 2$ evaluation:

$$- d(e_2) - \alpha\beta\rho d(e_3) \qquad\qquad -\beta\delta d(e_3)$$

At $t = 1$, the evaluated marginal disutility of effort at $t = 2$ is given by $-(\alpha\beta\rho + \beta\delta)d'(e_2)$, whereas the evaluated marginal disutility of effort at $t = 3$ is $-(\alpha\beta\rho^2 + \alpha\beta\delta\rho + \beta\delta^2)d'(e_3)$. At $t = 2$, the corresponding evaluated marginal disutility of $t = 2$ effort is now $-d'(e_2)$ versus evaluated marginal disutility of $t = 3$ effort of $-(\alpha\beta\rho + \beta\delta)d'(e_3)$. We assume that d is strictly convex so the subject would provide an interior solution for all moderate price ratios.[1]

For sake of argument, we now consider the case in which chores can be traded one-for-one between $t = 2$ and $t = 3$, and look for parameter combinations under which the $t = 1$ self would (under commitment) allocate an equal amount of effort to $t = 2$ and $t = 3$ or more effort to $t = 2$, but the $t = 2$ self faced with the same trade-off would allocate more effort to $t = 3$.[2] Mathematically, the $t = 1$ self would pick e_2 and e_3 to satisfy $d'(e_2) = \frac{\alpha\rho^2+\alpha\delta\rho+\delta^2}{\alpha\rho+\delta}d'(e_3)$. Thus, they would allocate equal efforts at $t = 1$ if $\alpha\rho^2 + \alpha\delta\rho + \delta^2 = \alpha\rho + \delta$. In contrast, the $t = 2$ self would set $d'(e_2) = (\alpha\beta\rho + \beta\delta)d'(e_3)$. They would set $e_2 < e_3$ if $\alpha\beta\rho + \beta\delta < 1$. However, our analysis requires the opposite inequality, $\alpha\beta\rho + \beta\delta \geq 1$, in order to explain an immediate completion tendency in our experiment. We conclude that this model of anticipatory utility with present bias is incapable of explaining both the immediate completion tendency we find and the evidence for present bias from CTB designs.

[1] This strict convexity assumption supported by the near-universality of interior allocations in Augenblick et al. (2015) as well as their parameter estimates.

[2] An equal effort allocation by the $t = 2$ self is an easy simplification, and is consistent with the estimated interpretation of the estimated $\delta \simeq 1$ by Augenblick et al. (2015). These two assumptions are consistent with both their structural estimates and their raw data.

Online Supplement B: Participant attrition

Table 1 documents the exact stopping point of the 101 participants who showed up to the an introduction of our experiment on a Friday focused on informed consent, instructions, and tech support.

Twelve participants who showed up to the Friday instructions did not begin the experiment on Monday. Seven of these explicitly dropped out during the Friday instructions and five more never started the experiment on Monday (despite receiving an email reminder). These twelve participants provided us with no choice data and we believe based on their comments and questions that many of them signed up for the experiment without carefully reading the required number of days or payment delay. Once they were fully aware of the requirements, these participants considered the fixed payment of $25 on Sunday to be insufficient.

These leaves seven participants who began the experiment on Monday but did not complete it.

Five of these seven stopped at points that we believe are best represented by forgetting or misunderstanding the instructions: the two who stopped signing in after their "extra chores done Tues" likely forgot or misunderstood given they had only one daily chore remaining; the two and one who stopped at "no decisions or chores Tues" and "No chore on Wed" respectively after signing in on those days may have left their daily requirements unfinished with an intention to return, but forgot.

The two who stopped participating between Monday and Tuesday ("No sign-in Tues") may be revealing their preferences as their Tuesday utility function manifests.

Total participants	101
Opt-out during Friday consent and instructions	7
No sign-in Mon	5
No sign-in Tues	2
No decisions or chores Tues	2
No sign-in Wed (extra chores done Tues)	1
No chore on Wed	1
No sign-in Thu (extra chores done Tues)	1
Completed all sign-ins, choices, and chores	82

Table 1: Participant attrition by experiment stage

Online Supplement C: Power analyses

Assume each participant in the population is one of three types (t = time-consistent, s = sophisticated, or n = naïve). We are interested in the power of our data to reject hypotheses of the form (P_t, P_s, P_n), where:

- P_t is the proportion of the population classified as time-consistent,
- P_s is the proportion of the population classified as sophisticated and not-time-consistent,
- P_n is the proportion of the population classified as naïve and not-time-consistent, and
- $P_t + P_s + P_n = 1$.

We report here on two measures of power:

1. Ex-post power – assuming our observed data to be the true population values of (P_t, P_s, P_n), what proportion of possible observations would fall outside the confidence region?
2. Ex-ante power – for any possible vector of proportions, would we reject the null hypothesis that our data were generated by that vector, and what proportion of possible observations fall outside the confidence region?

We follow the approach and terminology of Hall (1987). We construct bootstrap confidence regions that are likelihood-based (all parameter values inside the confidence region have higher likelihood than those outside).

Ex-post power

We treat our experiment data as the true proportion of types in the population and simulate $B = 10,000$ samples of $n = 52$ observations, indexed(P_t^b, P_s^b, P_n^b) for $b = 1, \ldots, B$.

A likelihood- based bootstrap confidence region with 95% significance level consists of the (convex hull of the) smallest number of observed values of (P_t^b, P_s^b, P_n^b) such that the region contains at least 9,500 observations (Hall, 1987). We acknowledge that

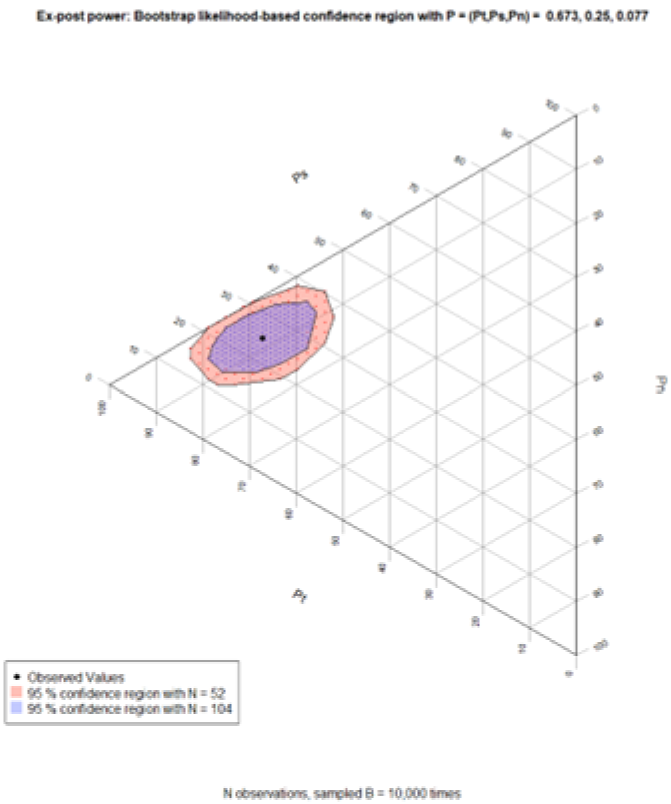

Figure 1: Confidence region for non-endogenous subsample data, n = 52 vs n = 104

this is not the smallest or fastest-converging bootstrapped confidence region covered in recent literature and that more modern and complicated confidence regions could converge to cover the true parameter more quickly. We chose these likelihood-based bootstrap confidence regions because they are easy to construct and explain and they converge to cover the true parameter with 95% probability (Hall, 1987). Recall our 52 non-endogenous experiment participants were classified as 35 time-consistent, 13 sophisticated, and 4 naïve, so we use probabilities of (0.673, 0.250, 0.077) in our simulations. To demonstrate the difference in power with a doubled sample size we repeat the process of B resamples assuming 104 instead of 52 observations per sample. The confidence region covers 6.5% and 3.1% of the parameter space under 52 and 104 observations, respectively. Figure 1 displays the two regions on the unit simplex. We forego the ability to reject a marginal 3.4% of the parameter space by limiting our sample to 52 non-endogenous participants versus doubling the sample to 104.

Ex-ante power

	Manhattan distance		Chebyshev distance	
	Mean	Sup	Mean	Sup
n = 52 participants	0.180	0.392	0.090	0.196
n = 104 participants	0.128	0.288	0.064	0.144

Table 2: Confidence region's largest distance from point prediction to outer hull for n = 52 vs. n = 104

There is a relatively small region of the parameter space in which there are null hypotheses we fail to reject with 52 observations that we would be able to reject by doubling our sample size to 104. To double our sample size would be costly, so here we demonstrate the marginal benefit of a doubled sample size by looking for null hypotheses that are only rejected with the greater power of a doubled sample, i.e., hypotheses where our experiment data lie within the red confidence region for 52 observations, but outside the blue confidence region for 104 observations. We construct bootstrapped confidence intervals for all values of $P_{t,}, P_s, P_n \in \{0.1, 0.2, \ldots, 0.9\}$ such that $P_t + P_s + P_n = 1$ and plot them in the 2-dimensional simplex.[3] This provides a sense of the regions of the simplex where we have power to reject null hypotheses, and where 104 observations could provide marginal power.

We acknowledge that there are a small set of null hypotheses, such as that pictured in Figure 2 that would be rejected had we observed the same data proportions with 104 participants. Specifically, Figure 2 shows we could reject the null hypothesis that$(P_t, P_s, P_n) = (0.6, 0.2, 0.2)$ with 104 observations, but we would fail to reject it with our current 52 observations. To estimate the extent of this power loss, for each non-degenerate null hypothesis we calculate the area of the parameter space that is covered by the 95% confidence region. The average confidence region covers 6.1% and 4.2% of the parameter space under 52 and 104 observations, respectively. We forego the ability to reject a marginal 1.9% of the parameter space by limiting our sample to 52 non-endogenous participants. An alternative measure of confidence region size is to consider the maximum distance from the null hypothesis to a point in the confidence region. We calculate the supremum of the Manhattan and Chebyshev distance for each null hypothesis in Table 2.

[3]Including degenerate distributions with $P_x \in \{0.0, 1.0\}$ for some x disingenuously improves the power as these parameters generate confidence intervals (instead of regions) with an area of zero.

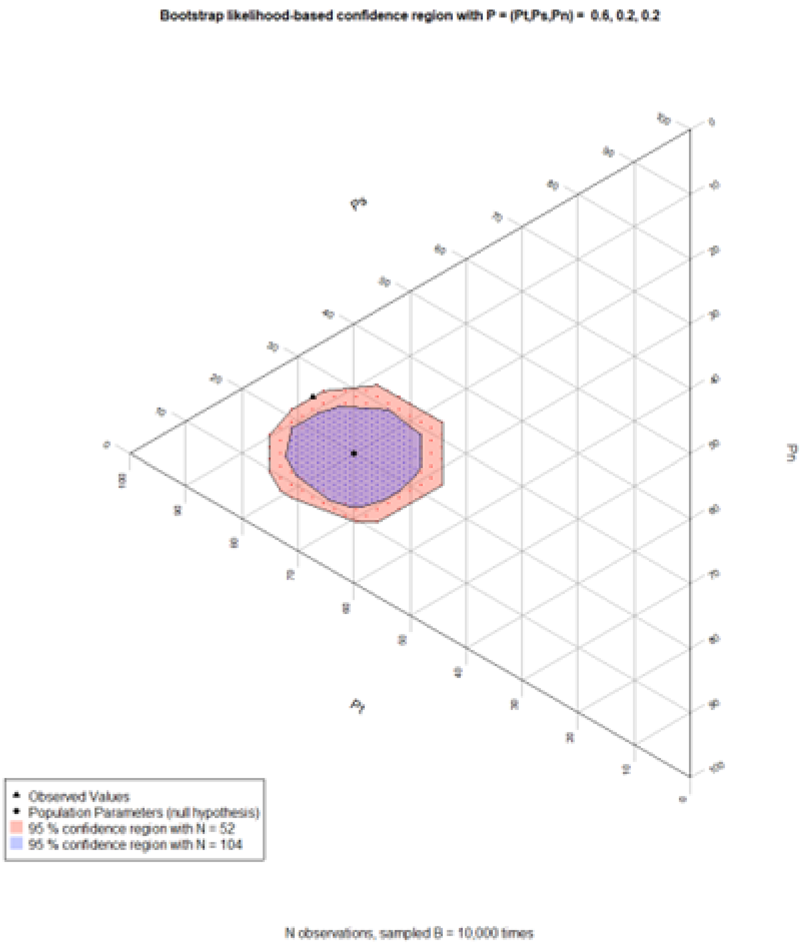

Figure 2: One separating case of hypothesis rejection for n = 52 vs. n = 104

Online Supplement D: Complete list of effort schedules by experiment version

	Version 1				Version 2				Version 3			
Schedule	Mon	Tues	Wed	Thurs	Mon	Tues	Wed	Thurs	Mon	Tues	Wed	Thurs
1	14	20	28	NA	14	20	28	NA				
2	14	20	NA	NA	14	20	NA	NA				
3	14	NA	28	NA	14	NA	28	NA				
4	NA	20	28	NA	NA	20	28	NA				
5	NA	14	20	28	NA	14	20	28				
6	NA	14	20	NA	NA	14	20	NA				
7	NA	14	NA	28	NA	14	NA	28				
8	NA	NA	20	28	NA	NA	20	28				
9	16	20	25	NA	16	20	25	NA	16	20	25	NA
10	16	20	NA	NA	16	20	NA	NA	16	20	NA	NA
11	16	NA	25	NA	16	NA	25	NA	16	NA	25	NA
12	NA	20	25	NA	NA	20	25	NA	NA	20	25	NA
13	NA	16	20	25	NA	16	20	25	NA	16	20	25
14	NA	16	20	NA	NA	16	20	NA	NA	16	20	NA
15	NA	16	NA	25	NA	16	NA	25	NA	16	NA	25
16	NA	NA	20	25	NA	NA	20	25	NA	NA	20	25
17	18	20	22	NA	18	20	22	NA	18	20	22	NA
18	18	20	NA	NA	18	20	NA	NA	18	20	NA	NA
19	18	NA	22	NA	18	NA	22	NA	18	NA	22	NA
20	NA	20	22	NA	NA	20	22	NA	NA	20	22	NA
21	NA	18	20	22	NA	18	20	22	NA	18	20	22
22	NA	18	20	NA	NA	18	20	NA	NA	18	20	NA
23	NA	18	NA	22	NA	18	NA	22	NA	18	NA	22
24	NA	NA	20	22	NA	NA	20	22	NA	NA	20	22
25	20	20	20	NA	20	20	20	NA	20	20	20	NA
26	20	20	NA	NA	20	20	NA	NA	20	20	NA	NA
27	20	NA	20	NA	20	NA	20	NA	20	NA	20	NA
28*	NA	20	20	NA	NA	20	20	NA	NA	20	20	NA
29	NA	20	20	20	NA	20	20	20	NA	20	20	20
30*	NA	20	20	NA	NA	20	20	NA	NA	20	20	NA
31	NA	20	NA	20	NA	20	NA	20	NA	20	NA	20
32	NA	NA	20	20	NA	NA	20	20	NA	NA	20	20

Table 3: Experiment effort schedules by version (part 1 of 2)
*Schedules 28 and 30 are identical, particpants see only one.

	Version 1				Version 2				Version 3			
Schedule	Mon	Tues	Wed	Thurs	Mon	Tues	Wed	Thurs	Mon	Tues	Wed	Thurs
33					19	20	21	NA				
34					19	20	NA	NA				
35					19	NA	21	NA				
36					NA	20	21	NA				
37					NA	19	20	21				
38					NA	19	20	NA				
39					NA	19	NA	21				
40					NA	NA	20	21				
41									22	20	18	NA
42									22	20	NA	NA
43									22	NA	18	NA
44									NA	20	18	NA
45									NA	22	20	18
46									NA	22	20	NA
47									NA	22	NA	18
48									NA	NA	20	18
49									25	20	16	NA
50									25	20	NA	NA
51									25	NA	16	NA
52									NA	20	16	NA
53									NA	25	20	16
54									NA	25	20	NA
55									NA	25	NA	16
56									NA	NA	20	16

Table 4: Experiment effort schedules by version (part 2 of 2)

References

Augenblick, N., Niederle, M., and Sprenger, C. (2015). Working over time: Dynamic inconsistency in real effort tasks. *Quarterly Journal of Economics*, 130(3):1067–1115.

Hall, P. (1987). On the bootstrap and likelihood-based confidence regions. *Biometrika*, 74(3):481–493.

Loewenstein, G. (1987). Anticipation and the valuation of delayed consumption. *Economic Journal*, 97(387):666–684.

Online Supplement E: Experiment instructions, consent, and procedure

ONLINE EXPERIMENT INSTRUCTIONS – 29 March 2021

Thank you for participating in this decision-making experiment. We will read through the instructions aloud together, and you may ask questions by using the chat in Zoom.

Basic Requirements and Payments

For showing up on time today, you will receive the show-up fee of $7 CAD by email transfer delivered to the email address you used to sign up for the experiment.

This experiment will require you to participate remotely from a computer or mobile phone on Monday, Tuesday, Wednesday, and Thursday next week at any time of your choosing.

If you sign in on all 4 days and complete the necessary decisions and tasks, you will be paid an additional $25 CAD by email transfer. If you miss one or more requirement, you will receive no additional payment, only your show up fee.

The email transfers will be sent after the experimenters have checked that you completed the necessary steps on Monday, Tuesday, Wednesday, and Thursday.

If you have not received your email transfer by 5pm the following ***Monday (5 April 2021),*** *please contact econexp@sfu.ca.*

Consent and Anonymity

The link below provides a consent form hosted through SFU's Web Survey. Please read it. If after reading the form, you would like to choose not to participate in the study you are free to leave. We will confirm consent forms are completed after the details of the study are discussed.

Your anonymity is assured. Your name is recorded only in the consent form in a separate database from the experiment, and so cannot be connected to the decisions you make. Your SFU IT username and associated SFU email will be used for signing in, to receive reminders, and to receive money transfers when you have completed all requirements for the week. Immediately following your last payment for the study, the record of your email will be destroyed and will not be connected to your responses in the study.

All participants please sign in at the following link to view the consent form now

http://websurvey.sfu.ca/survey/378337633

Overview of Experiment

This is a study about your willingness to perform a number of transcription tasks (chores) now versus later. Today, you will learn about the study and practice signing in and doing the chore on your own internet connected device.

There are four (4) more participation dates that occur remotely online on Monday, Tuesday, Wednesday, and Thursday next week (29 March – 1 April 2021).

On each of the participation dates, you must sign in, make decisions, and complete chores at any time of your choosing between 00:00 and 23:59. All requirements for a day (sign-in, decisions, and chores) must be complete by 23:59 on that day to be eligible for payment at the end of the week. Payment will be made by email transfer by the Monday that follows your last requirement.

Chores: Greek Transcription Task

The chore involves transcribing a line of 40 blurry letters of Greek text.

In the chore, a string of blurry Greek text will appear in a Transcription Box on your screen. For each blurry character you will need to find and select the corresponding character and enter it into the Completion Box by clicking the appropriate button on your screen. Examples will follow.

One chore is one line of Greek text. For a task to be complete your accuracy must be 100%. If you submit an inaccurate line of text, you will have to start the chore over again.

Each day that you are required to sign in, you must complete a minimum of one chore.

On one (and only one) of the days of the week, you must also complete a number of extra chores. The number of extra chores can vary by day and across participants.

Each day you sign in, if you have not completed the extra chores for the week yet, you will be in control of whether you want to do extra chores Today or Not Today.

Today/Not Today Decisions

After you sign in on Monday, you will be displayed a list of chore schedules, and for each one you must choose whether you would prefer to work Today or Not Today.

A chore schedule describes your options to complete the extra chores required to earn your $25 payment for the week. For example, a single chore schedule might look like Figure 1:

Figure 1

Schedule No.	Chores if done Mon (Today)	Chores if done Tue	Chores if done Wed	Chores if done Thu	Choices (Pick One)	
					Today	Not Today
1	16	20	25	NA	○	○

Only one of the chore schedules in the list will be randomly selected by the computer with equal probability to be the relevant schedule for payment.

You will not receive any information on which is the relevant schedule until after you have made a (Today/ Not Today) decision for each schedule in the list. Because there is a positive and equal chance of any schedule in the list being the relevant schedule for your payment, you should make each decision carefully in your best interest as if it is the relevant schedule for your payment.

Supposing the schedule in Figure 1 is chosen as the relevant schedule, a subject is eligible for payment if she completes **16** extra chores on Monday OR **20** extra chores on Tuesday OR **25** extra chores on Wednesday. She only needs to complete the extra chores on one of the three days to complete the relevant schedule. Thursday is **(NA)** not available for this chore schedule, so if this is the relevant schedule and you do not complete your extra chores Monday or Tuesday, you must complete them on Wednesday to be eligible for your extra payment.

Example 1

On Monday, Subject A signs in and can see her completion status for the week, as in Figure 2:

Figure 2

Today is a game day!

Thanks for signing in.

TODAY	MON 06 JUL
CURRENT WEEK	MON 06 JUL- SUN 12 JUL
WEEK NUMBER	1 OUT OF 1
MINIMUM CHORES FOR TODAY	1
PAYOFF FOR COMPLETING WEEK'S REQUIREMENTS	$20
PAYOFF DATE	MON 13 JUL
YOUR EXPERIMENT ID	guk1r7qb

	PAYOFF REQUIREMENT	COMPLETION STATUS
1	SIGN IN 4 TIMES (MONDAY, TUESDAY, WEDNESDAY AND THURSDAY)	1/4 COMPLETE
2	MAKE "TODAY" / "NOT TODAY" WORK DECISIONS **MAX** 3 TIMES (MONDAY, TUESDAY AND WEDNESDAY)	0/3 COMPLETE
3	PERFORM MINIMUM CHORES 4 TIMES (MONDAY, TUESDAY, WEDNESDAY AND THURSDAY)	0/4 COMPLETE
4	PERFORM EXTRA CHORES 1 TIME (MONDAY, TUESDAY, WEDNESDAY OR THURSDAY)	0/1 COMPLETE
5	OVERALL THIS WEEK	NOT COMPLETE
6	WEEK NUMBER	1 OUT OF 1 WEEK(S)

On the next page, Subject A is presented the list of chore schedules below. She enters her Today / Not Today decisions for each schedule by clicking the corresponding button on the right. Note there is no choice to be made if doing chores today is not available (NA). Her decisions are included below:

Figure 3

Schedule No.	Chores if done Mon (Today)	Chores if done Tue	Chores if done Wed	Chores if done Thu	Choices (Pick One)	
					Today	Not Today
1	16	20	25	NA	◉	○
2	NA	20	25	NA	○	◉
3	16	NA	25	NA	◉	○
4	16	20	NA	NA	◉	○
5	20	22	23	NA	○	◉
6	NA	22	23	NA	○	◉

After clicking 'Submit', she is displayed the following screen:

Figure 4

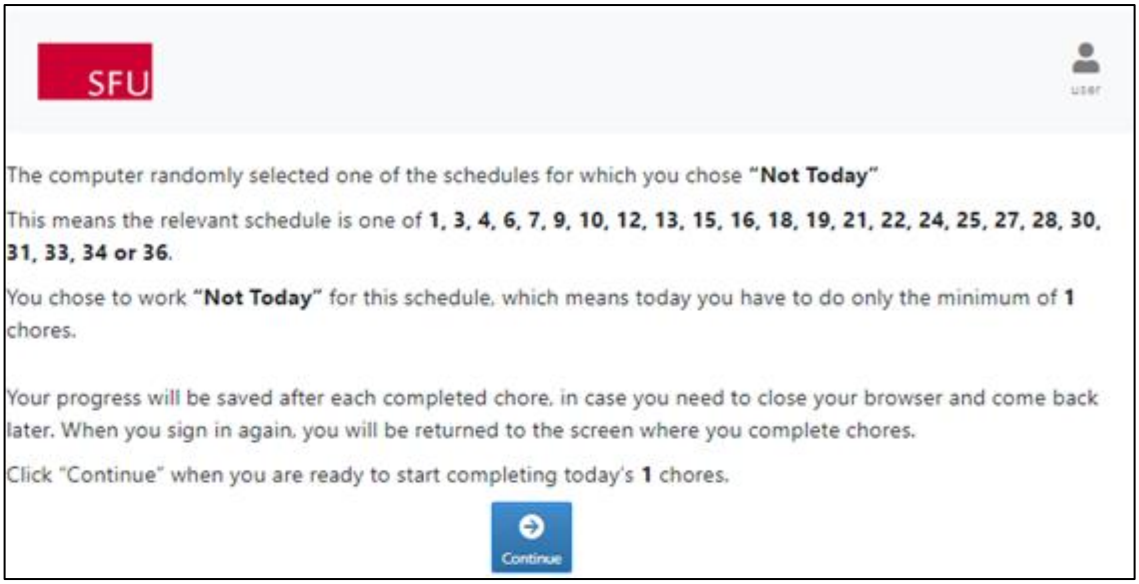

The next screen is the chore page, on which Subject A completes her 1 required chore for Monday.

Figure 5

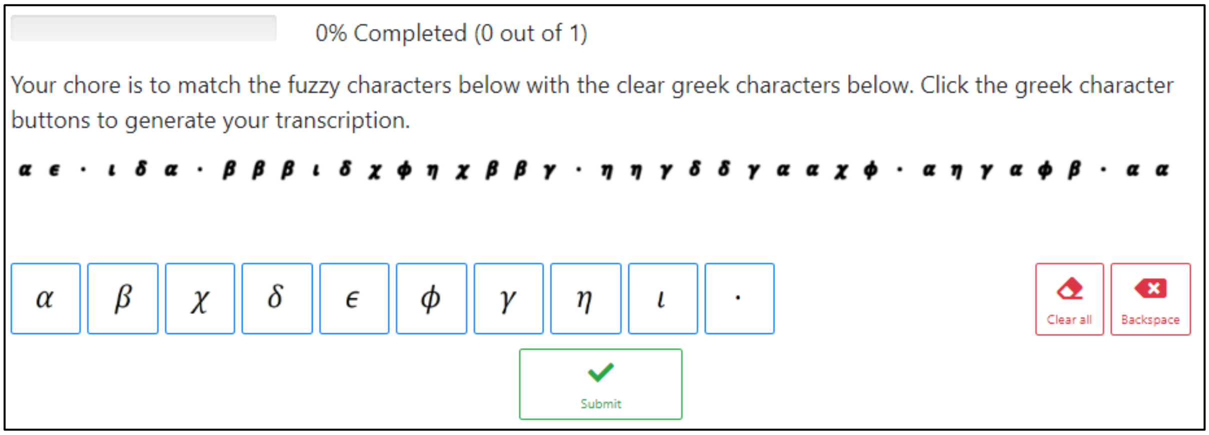

After the chore is complete, Subject A is shown a revised completion table and is finished for the day.

Tuesday: When Subject A signs in on Tuesday she now sees a revised list of chore schedules (because the option to do extra chores on Monday is no longer available). But she still has to make a Today/Not Today choice for each unique schedule. If two schedules are identical only one will be displayed, so every decision you are asked to make is different. This is why Schedule 2 no longer appears in Figure 6, because Schedule 2 is the same as Schedule No 1 on all days except Monday.

Figure 6

Schedule No.	Chores if done Tue (today)	Chores if done Wed	Chores if done Thu	Choices (Pick One)	
				Today	Not Today
1	20	25	NA	○	○
3	NA	25	NA	○	○
4	20	NA	NA	○	○

Just like on Monday, after clicking 'Submit' the subject is informed of whether she chose Today or Not Today for the relevant chore schedule, and the number of chores required Today is displayed.

<u>Example 2</u>

On Monday, Subject B is presented the list of chore schedules below. She enters her Today / Not Today decisions for each schedule by clicking the corresponding button to the right of the chore schedule. Her decisions are included below:

Figure 7

Schedule No.	Chores if done Mon (Today)	Chores if done Tue	Chores if done Wed	Chores if done Thu	Choices (Pick One)	
					Today	Not Today
1	16	20	25	NA	(•)	( )
2	NA	20	25	NA	( )	(•)
3	16	NA	25	NA	(•)	( )
4	16	20	NA	NA	(•)	( )
5	20	22	23	NA	( )	(•)
6	NA	22	23	NA	( )	(•)

After clicking 'Submit', she is displayed the following screen:

Figure 8

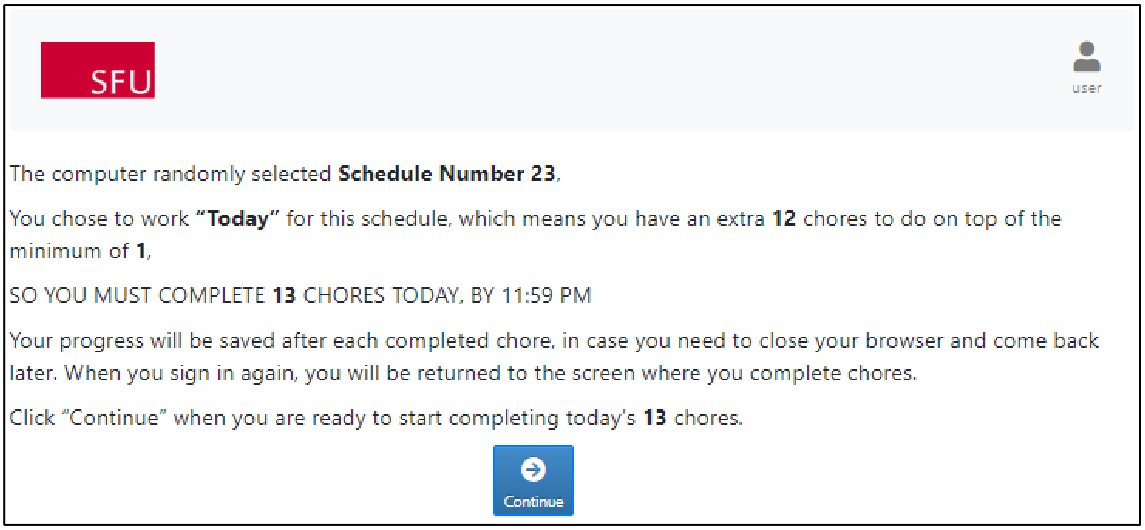

Completion Table

After you sign in and before you sign out each day, a table of your completion status is displayed to remind you of what still needs to be done to be eligible for the $25 payment at the end of the week. An example completion table is in Figure 9:

Figure 9

Thank you for your continued participation in the experiment.

Today you completed **13** chores, the minimum of **1** plus **12** chores from the relevant effort schedule.

Please review the revised completion table below.

	PAYOFF REQUIREMENT	COMPLETION STATUS
1	SIGN IN 4 TIMES (MONDAY, TUESDAY, WEDNESDAY AND THURSDAY)	1/4 COMPLETE
2	MAKE "TODAY" / "NOT TODAY" WORK DECISIONS **MAX** 3 TIMES (MONDAY, TUESDAY AND WEDNESDAY)	1/3 COMPLETE
3	PERFORM MINIMUM CHORES 4 TIMES (MONDAY, TUESDAY, WEDNESDAY AND THURSDAY)	1/4 COMPLETE
4	PERFORM EXTRA CHORES 1 TIME (MONDAY, TUESDAY, WEDNESDAY OR THURSDAY)	1/1 COMPLETE
5	OVERALL THIS WEEK	NOT COMPLETE
6	WEEK NUMBER	1 OUT OF 1 WEEK(S)

Your next required sign-in is **Tues 07 Jul** between 12:00am and 11:59pm.

An email/text will be sent at your chosen time as a reminder to sign in and complete your decisions and chores for payment

Experiment for Payment Begins Monday 29 March 2021

Sign in on Monday and use the link *"29March2021_Experiment"* to begin the experiment for payment.

Email Reminders

On the first day of the experiment (Monday) you will be required to select a time to receive email reminders on work days.

At any time throughout the experiment, you can sign in and adjust the timing of your reminders.

Issues

Please describe any technical issues you face with screenshots in an email to econexp@sfu.ca.

Consent and Practice Round

We will now complete a practice round as a group. This will allow you to ask questions as you experience each page of the experiment on your own device. The practice round has only one extra chore which must be completed today, so you will do the minimum (1) chore plus (1) extra chore in the practice round now. After you complete two total chores you have completed the requirements for today.

NOW: Please sign-in to the experiment page to complete the Practice Round:

The sign-in system works best if you use Google Chrome on Incognito Mode

https://lab.econ.sfu.ca/oScope/participate

Choose the link that says *"26March2021_Practice"*

MONDAY: The Experiment will be accessible at 12:00am Monday 29 March 2021

The sign-in system works best if you use Google Chrome on Incognito Mode

https://lab.econ.sfu.ca/oScope/participate

Choose the link that says *"29March2021_Experiment"*

SFU

CONSENT FOR PARTICIPATION IN SOCIAL AND BEHAVIOURAL RESEARCH **2016s0245**

Description: All participants have been recruited via the online recruiter system of the SFU Experimental Economics Lab. The purpose of this experiment is to study economic decision-making. This orientation session will last about 30 minutes and will prepare you to participate in an online experiment on Monday, Wednesday, and Friday of next week. As a participant in our recruiter database, you will be regularly invited to participate in economics experiments organized by the Lab.

Risks and Benefits: There are no foreseeable risks to you from participation in this experiment. The primary benefit of the study will be to advance understanding of economic decision-making processes.

Cost and Payments: You will receive a cash payment of $7 for the orientation session today. In addition to this payment, you will also receive a monetary payment through an electronic transfer of $20 on Saturday of next week if you complete all requirements of the online experiment on Monday, Wednesday, and Friday of next week. There are no costs to participating in the study.

Confidentiality: You understand that any information about you obtained from this research will be kept confidential. All electronic data collected will be anonymized so that records cannot be matched to individual participants. Data will be collected on a server, but it will be transferred to the PI's computer and deleted from the server at the conclusion of the experiment. Any information that could be used to personally identify you (which will consist only of signed receipts) will be stored in a locked filing cabinet in the locked office of the PI with access limited to the PI and his authorized research assistants, all of whom have signed statements agreeing to keep this information confidential. It has been explained to you that your identity will not be revealed without your consent in any description or publication of this research, and the data will be made anonymous after collection so that your identity cannot be tied to your decisions. Moreover, you understand that the anonymous data will be preserved indefinitely while receipts will be preserved until 2023. Therefore, you consent to such publication for scientific purposes.

Right to Refuse or to End Participation: You understand that you are free to refuse to participate at any time and that your decision will not expose you to any penalty or loss of benefits to which you might be otherwise entitled but will entail loss of all earnings from the experiment itself, except for the participation fee. You understand that such a decision will not adversely affect your standing in any class in which you are enrolled. You also understand that you may be withdrawn from the study at any time by the investigators.

Availability of Research Results: You understand that the results of the research in which you are participating will be publicly available through the website of the primary investigator (http://www.sfu.ca/~dfa19).

SFU

CONSENT FOR PARTICIPATION
IN
SOCIAL AND BEHAVIOURAL RESEARCH

I consent to participating in research entitled: Experimental Studies of Economic Decision Making.

David Freeman (Principal Investigator, Department of Economics) or his/her authorized representative has explained the purpose of the study, the procedures to be followed, and the expected duration of my participation. Possible benefits of the study have been described, as have alternative procedures, if such procedures are applicable and available.

I acknowledge that I have had the opportunity to obtain additional information regarding the study and that any questions I have raised have been answered to my full satisfaction.

Furthermore, I understand that I am free to withdraw consent at any time and to discontinue participation in the study without prejudice to me.

I have had a chance to ask questions and to obtain answers to my questions. I can contact the investigator at 778-782-9634 or david_freeman@sfu.ca. If I have questions about my rights as a research participant or I have any concerns or complaints about the research, I can contact Dr. Jeff Toward, Director, Office of Research Ethics at jtoward@sfu.ca or 778-782-6593.

Finally, I acknowledge that I have read and fully understand the consent form. I sign it freely and voluntarily. A copy has been given to me.

Date: ______/____/____
yyyy/mm/dd

Subject's Name (**printed please**) ______________________________

Subject's **Signature**: _____________________________

Subject's SFU e-mail address ___________________

Signed:___________________________
(Principal Investigator or his/her authorized representative)

Online Experiment Procedure

1) Recruiting: Participants registered to the Economics Department's online recruitment system are randomly selected to receive a recruitment email to the study, with the following text:

> Dear %FIRST_NAME%,
>
> You are invited to participate in an economics experiment.
>
> An orientation session for the experiment will take place online on %INTRO_FRIDAY% at %INTRO_TIME% via Zoom.
>
> The experiment will require you to make decisions and/or complete tasks through an online interface on each of Monday, Tuesday, Wednesday, and Thursday (%DATES%) of the following week.
>
> To earn full payment for the experiment, you must attend the orientation session and complete all requirements on all these days.
>
> All payments will be made by e-transfer and requires that you have a Canadian bank account.
>
> To participate, please sign up through your account at sfu-experiments.sona-systems.com.
>
> Thank you for your interest in economics experiments!
>
> -SFU Experimental Economics Lab

2) Participants register online, with a registration cut-off time approximately 24 hours before the scheduled experiment start.

3) After registration deadline, experimenter uploads the Central Authentication Service usernames of registered participants, restricting experiment access to registered users.

4) Experimenter creates a password-protected Zoom meeting beginning 30 minutes before the schedule start time.

5) Experimenter emails participants who registered through Sona-systems recruiting software, with the following text:

You are scheduled to participate in the study 'Experiment on Decision-Making III' on %INTRO_FRIDAY% at %INTRO_TIME% via Zoom

%ZOOM_LINK%

The researchers are: %RESEARCHER_NAMES%

Please join the Zoom meeting between %INTRO_TIME_OPEN% and %INTRO_TIME%.

Please be prepared to show SFU ID.

All participants who arrive before %INTRO_TIME%. will be permitted to enter one-by-one and participate.

If you arrive at the zoom waiting room after %INTRO_TIME%. you may not be permitted to enter.

Best

%RESEARCHER_NAMES%

6) Experimenter admits students to the meeting one at a time.
   a. Check their ID by camera
   b. Enter student attendance in online reservation system
   c. Move student to breakout room, where they remain for most of experiment
   d. Send email to students in attendance via econ_experiments@sfu.ca with pdf instructions and consent form link:

FIRST, please read the consent form at the following link:

http://websurvey.sfu.ca/survey/378337633

We will read the attached experiment instructions together and take questions.

Do not distribute the experiment instructions to anyone and do not post them anywhere.

%RESEARCHER_NAMES%

%RESEARCHER_EMAIL%

7) When participants have completed the consent form, Experimenter shares the experiment link to all participants. The link first directs them to the University's Central Authentication Service, and once their identity is confirmed, to the experiment landing page, which begins with instructions.

8) Experimenter reads instructions aloud while sharing instructions on screen
    a. The emailed pdf instructions are identical to those at the beginning of the online experiment.

9) Participants with questions are instructed to use the 'Raise Hand' function in Zoom. For each student question, the experimenter removed the participant from the breakout room and answer question one-on-one in the main room. Then add them back to the breakout room.

10) Participants are asked to confirm they can sign-in to the experiment link. Participants are asked to confirm with the Experimenter that they have completed one chore before being dismissed from the Zoom meeting. Experiment provides support until all participants are successful.
